# Political economics in health and implications for neurosurgery diseases

**DOI:** 10.3389/fpubh.2024.1444249

**Published:** 2025-01-28

**Authors:** Yi Han, Yutao Huang

**Affiliations:** ^1^School of Economics and Management, Leshan Normal University, Leshan, China; ^2^Department of Neurosurgery, West China Hospital, West China Medical School, Sichuan University, Chengdu, China

**Keywords:** political economics, public health, neurosurgery, TBI, stroke, ICH, brain tumor

## Abstract

The field of political economics in health has a significant and far-reaching impact on public health. It encompasses a diverse range of interconnected domains, including the economy, welfare, the environment, food and drug safety, pollution emissions, occupational safety, the quality of medical services, consumer rights, public health policy, healthcare policy, scientific research, and marketing management. In this review, we examine the global influence of political economics on health outcomes and delineate the impact of prevalent neurosurgical conditions on individual and collective healthcare resources. This review will discuss the effects of political-economic factors on the prevalence and treatment of neurosurgical diseases, including stroke, traumatic brain injury (TBI), intracerebral hemorrhage (ICH), and brain malignant tumors. Furthermore, the current challenges and future directions will be discussed. We intend this review to facilitate the exchange and integration of political economics, public health, and neurosurgery, provide a foundation for policy development, enhance the prevention, diagnosis, and treatment of neurosurgical diseases, and ultimately promote public health.

## 1 Introduction

Political economics is an interdisciplinary field of study that draws upon political science, economics, and sociology disciplines. Its objective is to comprehend the interconnections between economic systems, political institutions, and social structures. The field’s core topics encompass resource allocation, power and interests, institutions and policies, globalization and international relations, and other related areas. Political economics in health is a subfield of political economics that concentrates on the political, economic, and social elements influencing public health concerns. Its objective is to comprehend and address the intricate issues affecting public health and to formulate evidence- and science-based policy recommendations to enhance global and local health outcomes. The field’s central concerns pertain to the influence of political-economic systems, regulatory frameworks, policies, and commercial entities on public health. The political-economic system is primarily concerned with the economy, social welfare, and the environment. On the contrary, the regulatory system, is primarily focused on food and drug safety, pollution emissions, occupational safety, the quality of medical services, and consumer rights. The domain of policies encompasses both public health policies and healthcare policies. Meanwhile, business entities are inextricably linked to scientific research and marketing management. Consequently, political economy exerts a principal influence on public health through these channels.

Neurosurgery represents a significant subspecialty of surgery and a prominent public health concern. The most common neurosurgical diseases are traumatic brain injury (TBI), stroke, intracerebral hemorrhage (ICH), and brain malignant tumors. These conditions significantly burden family and social healthcare resources and present a substantial challenge to public health. TBI represents a significant global public health concern, with over 50 million individuals worldwide affected annually, resulting in economic losses estimated at up to $400 billion (1, 2). TBI is the leading cause of death in young people and the predominant reason for death and disability at all ages in countries across the globe. Approximately half of the global population is estimated to experience a TBI at some point during their lifetime (1). Stroke represents a significant global public health concern. It is estimated that 101 million individuals worldwide are afflicted with stroke, resulting in 6.55 million deaths (3). Overall, from 1990 to 2019, there was a 70.0% increase in the incidence of stroke, an 85.0% increase in the prevalence of stroke, and a 43.0% increase in the number of stroke deaths (3). The global incidence of ICH is estimated to be between 10 and 20 cases per 100,000 individuals, with an observed increase with age (4). The prognosis of ICH is unfavorable, with 7-day and 1-year mortality rates reaching as high as 31 and 59%, respectively. This places a significant burden on both the individual and their family (5). Malignant brain tumors can be classified into two main categories: primary brain tumors and metastatic brain tumors. According to the latest data, gliomas represent over 80% of primary malignant brain tumors, with an incidence rate of approximately 7/100,000, which is rising with age (6). Although gliomas have a relatively low incidence compared to other conditions such as TBI and stroke, they have a poor prognosis and are costly to treat. This often results in a significant financial burden on individuals and families. The incidence of brain metastases resulting from the growth of malignant tumors is approximately 9.6% (7). Nevertheless, the prognosis for patients with brain metastases from a tumor is exceedingly poor, resulting in a significant escalation in treatment costs (8). Treating these diseases requires a significant investment of medical resources and places a considerable burden on families and society.

The field of public health has effectively applied the tenets of political economy. Given its status as a significant area of public health, neurosurgery merits greater attention from political economists. This field of study can provide insights that inform public health activities, including disease prevention, treatment, research, and data collection. The incidence of TBI, stroke, and ICH can be reduced by implementing legislative and policy instruments, such as the enacting strict traffic safety regulations, establishing workplace safety standards, and introducing protective measures for sports. Moreover, the government and non-governmental organizations should reinforce public education and awareness campaigns on preventing neurosurgical diseases, promote educational activities on healthy lifestyles, and enhance public knowledge of the risks of neurosurgical diseases and protective measures. Political economics underscores the importance of equity and efficiency in the distribution of resources. This is of particular importance about the treatment and rehabilitation of neurosurgical diseases. Furthermore, political economics also emphasizes the importance of innovation and scientific and technological progress in the field of medical development. It is recommended that the government allocate greater financial resources to support fundamental and clinical research initiatives related to neurosurgical disorders, while concurrently facilitating the advancement of innovative therapeutic modalities and technological advancements. Furthermore, the field of political economics underscores the significance of data in informing decision-making processes. The establishment of a data collection and monitoring system for neurosurgery diseases, incorporating electronic medical records, social media, patient summaries, genomic and drug data, clinical trials, telemedicine, mobile app, behavioral and socio-economic indicators, and other relevant information, would facilitate the generation of an accurate and comprehensive picture of the incidence, treatment effects, and recovery outcomes associated with neurosurgery diseases. Furthermore, such a system would provide a scientific foundation for evidence-based policymaking.

Political-economic decisions in healthcare are confronted with many challenges, including the allocation of resources, the impact of globalization, the scientific basis of policy, strategic planning, the protection of privacy, and the resolution of ethical issues. It is of the utmost importance to determine how to confront these issues and propose solutions to advance the field of political economics in health. Improved communication between policymakers, healthcare workers, economists, and other professionals is essential. The decision-making process in political economics regarding healthcare inevitably involves a complex interplay of competing interests, scarce resources, and evolving social contexts. This inherent complexity places significant demands on policymakers.

This review examines the global impact of political economics on public health, which encompasses a range of interrelated domains, including the economy, social welfare, the environment, food and drug safety, pollution emissions, occupational safety, the quality of medical services, consumer rights, public health policy, healthcare policy, scientific research, and marketing management. In addition, this review presents an overview of the impact of common neurosurgery diseases (TBI, stroke, ICH, and brain malignant tumor) on national and state healthcare resources. Furthermore, it examines the implications of political economics in health on neurosurgery diseases, the current challenges encountered, and potential future directions for solutions. In general, the developed countries’ development experiences and conclusions are more pertinent and applicable. However, the development experience of some of the more populous developing countries is also very useful, which is why countries such as China, India, and Indonesia are included in the analysis. In particular, China, as the world’s second-largest economy, is also one of the world’s most populous countries. As a developing country, China has developed rapidly in recent decades, and its experience is therefore very worthwhile for some developing countries to learn from, which is why we have mentioned the Chinese experience many times. We hope this review will facilitate the exchange and integration of political economics, public health, and neurosurgery, thereby providing a foundation for policy development and ultimately enhancing public health outcomes.

## 2 Political economics in health

Political economics in health is an interdisciplinary field of study that examines the economic and political factors contributing to health issues. The field of political economics in health examines the impact of political-economic systems, regulatory frameworks, policies, and commercial entities on the health status of populations and health inequalities. This encompasses key economic, social welfare, and other factors. The following areas are also included: the environment, food and drug security, pollutant emissions, occupational safety, the quality of medical services, consumer rights, public health policy, healthcare policy, scientific research, and marketing management (Table 1). The field of political economics in health posits that the determinants of health are not solely biomedical, but rather are embedded within the intricate tapestry of socio-economic and political structures. Understanding these factors can facilitate the development of more effective health policies and interventions, which may in turn lead to a reduction in health inequalities and an improvement in society’s overall health.

**Table 1:** Political economics in health.

Political-economic field	Related to	Description	References
Economy	Greece	Prevalence of COPD rose to 18.2% from 5.6% because of the financial crisis in 2009	(16, 17)
	United States	Decreased socio-economic status leads to health problems during COVID-19 pandemic	(18)
	United Kingdom	Dropped financial budget in healthcare leads to excessive diseases	(19, 20)
	China	Healthcare infrastructure was improved due to economic growth and regional integration	(21)
Welfare	China	BCSIP improved people’s health and saved 18.8% on healthcare costs by improving digital infrastructure	(24)
	United Kingdom	Decreased welfare causes health problems	(25)
	Indonesia	Universal healthcare coverage causes a significant decline in neonatal and child mortality rates	(27)
	Global	Children’s deaths decreased because of improvements in women’s education levels	(29)
Environment	El Salvador	El Salvador rejected the Australian–Canadian conglomerate Oceana Gold from mining gold in the country due to the pollution of water resources caused by gold exploration and mining	(34)
	Global	Paris Agreement aims to coordinate national environmental policies to reduce CO_2_ emissions	(35)
	EU	EU has a world-leading policy system for environmental protection	(39, 40)
	China	China promotes economic transformation and environmental protection through the implementation of strict environmental laws, large-scale afforestation, and investment in clean energy	(41)
	United States	The United States government exited and rejoined the Paris Agreement	(42, 43)
Food and drug security	United States	FSMA was introduced in the United States to reduce foodborne illness	(67)
	Global	FDA ensured food and drug security	(68, 69)
	United States	FDA issued two messages addressing critical new warnings related to products containing the sedative/hypnotic zolpidem	(71)
	United States	FDA required manufacturers to add a warning to the labels of antidepressant medications regarding the risk of suicide in the pediatric population	(72)
Pollutant emission	China	TCZ policy which aimed to regulate the emission of SO_2_ from factories, control acid rain, and improve air quality has made respiratory morbidity declined	(73)
	China	China’s regulation of fireworks displays has reduced PM2.5 emissions and mitigated air pollution, thereby improving public health and reducing the incidence of respiratory and cardiovascular diseases	(74)
	China	CES which aimed to increase the use of clean energy and reduce air pollution has improved people’s health	(77)
	Global	BCCTMHWD and WEEED forced producers, sellers, and consumers to share the cost of disposal to reduce pollution	(79, 80)
Occupational safety	United States	OSHA has contributed to a significant reduction in work-related deaths, injuries, and illnesses	(82, 83)
	United States	Safe training protects workers’ health	(84, 85)
	New Zealand	Provision of sun protection equipment for workers working outdoors reduces sunburn to the skin from ultraviolet rays and reduces the risk of skin cancer	(87)
	United Kingdom	Shift work systems not only protect employee safety but also improve productivity	(90)
Quality of medical service	United States	NIH assists CVISU in offering a variety of training programs about the latest research techniques and mechanisms, improving the quality of medical therapy	(92)
	EU	EMAPC assists the EU in adopting the ‘Pediatric Regulation’, which forces pharmaceutical companies to monitor indications and adverse reactions of pediatric medicines and improves drug safety	(93)
	WHO	WHO brought new guidelines to the classification, diagnosis, and treatment of CNS tumors	(95, 96)
	Ignaz Semmelweis	Handwashing was incorporated into healthcare	(97)
	United States	NYSCSRS improves cardiovascular surgery by collecting clinical data from hospitals	(98)
	United Kingdom	A study suggested that organizations subject to complaints need to be aligned with the complainant and that the handling of complaints can be enhanced through linguistic analysis and reference to the consumer literature’s justice-based approach	(100)
Consumer right	India	CPSPR ensures product safety and consumer right	(101)
	United States	PPHPPSA aims to reduce the levels of toxic chemicals in consumer products, improving product safety and protecting people’s health	(104)
	China	CCCPC and the State Council announced DDERCPQE to call on thewhole people to be more active in strengthening structural reform of the education system, comprehensively promoting quality education	(107)
Public health policy	Global	Mass vaccination policies were adopted to control serious infectious diseases	(109, 110)
	China	Complete lockdown and quarantine measures were implemented to control the outbreak	(116)
	Global	Government changed individuals’ actions and guided their medication through various methods	(117)
Healthcare policy	United States	ACA reduced health inequalities and improved the accessibility of healthcare services	(118)
	United States	Government promotes innovative medical service models such as family doctor systems, telemedicine services, and short-term medical services, to meet diversified medical needs	(119–121)
	United States	Large-scale public policy reduced the incidence of disease and mortality rates	(125)
	The Netherlands	Government encourages people to adopt a healthy lifestyle through health education and health promotion policies	(126)
Scientific research	BioNTech SE and Pfizer Inc.	BNT162b2 vaccine has significantly prevented the spread of COVID-19	(127)
	Philips Healthcare (Koninklijke Philips N.V.)	eICUCRD covers medical data from 208 hospitals in the United States on more than 200,000 cases to improve the management of critically ill patients and to inform policy development in the health sector	(128)
	Symphony natural health (Natural Health International (Sales & Distribution) Inc.)	Melatonin is an effective antioxidant, immune activator, and mitochondrial modulator as a dietary supplement	(129)
	GlaxoSmithKline (GSK plc.), Eli Lilly (Eli Lilly and Company), and Abbott (Abbott Laboratories)	Several commercial entities are engaged in environmental impact studies to explore how to reduce the adverse effects of environmental pollution, e.g., carbon emission	(130, 131)
	Merck (Merck & Co., Inc.)	Merck initiated a large clinical trial on the anti-inflammatory drug rofecoxib (Vioxx) and misrepresented the results to hide evidence of rofecoxib’s cardiovascular toxicity, which led to thousands of cardiovascular events that should be avoided in patients taking the drug	(132)
Marketing management	Global	Tobacco companies continue to market in a variety of ways that influence smoking behavior	(134)
	Poland	The weakening of policies on restricting alcohol consumption accompanied by a large number of advertisements for beer led to an increase in alcohol-related mortality	(136)
	Global	Medicines did not contain enough active ingredients were marketed, leading to several diseases	(137)

ACA, Affordable Care Act; BCCTMHWD, Basel Convention on the Control of Transboundary Movements of Hazardous Wastes and their Disposal; BCSIP, Broadband China Strategy and Implementation Plan; CCCPC, Central Committee of the Communist Party of China; CES, Clean Energy Strategy; CNS, central nervous system; CPSPR, Consumer Product Safety Policy and Regulation; CVISU, Cardiovascular Institute at Stanford University; COPD, chronic obstructive pulmonary disease; DDERCPQE, Decision on Deepening Educational Reform and Comprehensively Promoting Quality Education; eICUCRD, eICU-Collaborative Research Database; EMAPC, European Medicines Agency’s Pediatric Committee; EU, The European Union; FDA, Food and Drug Administration; FSMA, Food Safety Modernization Act; NIH, National Institutes of Health; NYSCSRS, New York State Cardiac Surgery Reporting System; OSHA, Occupational Safety and Health Administration; PPHPPSA, Pollution Prevention for Healthy People and Puget Sound Act; TCZ, Two Control Zones; United Kingdom, The United Kingdom; United States, The United States; WEEED, Waste of Electrical and Electronic Equipment Directive; WHO, World Health Organization.

### 2.1 Political-economic system

The political-economic system is defined as the system in which a countries or region’s economic structure and political organization interact. It encompasses the impact of the government system, market mechanism, social organization, laws and regulations, and international relations on the country’s economy, welfare, and environment. A country’s political-economic system exerts both direct and indirect effects on the health status of its population (9). Moreover, a country’s political and -economic system serves as the foundation for its economic development, which, in turn, directly impact the purchasing power, living standards, living conditions, and the medical environment of its people. Consequently, the health of the population is influenced by these factors. Next, a country’s political-economic system determines the level of infrastructure development and public service provision. The capacity of the government to invest funds and resources in the provision of basic medical services, clean water, and sanitation facilities has a direct impact on the level of health experienced by the population. Inadequate or uneven distribution of public services and infrastructure may result from political-economic instability or imperfections, which can ultimately contribute to widening of social inequalities and health disparities (10, 11). The social determinants of health, including poverty, unemployment, and social exclusion, have been demonstrated to influence an individual’s health status. For example, the availability of universal healthcare coverage, the level of access to education, and the robustness of social assistance systems can profoundly impact on the population’s health (12). Furthermore, the political-economic system is intertwined with environmental management, which directly impacts human health. The government’s legislative and regulatory actions regarding the environment significantly impact the control of pollutant emissions, the optimization of resource utilization, and the enhancement of air quality. Accordingly, a healthy political-economic system should be one that can provide basic public services, safeguard economic stability and development, implement sound social welfare policies, and attach importance to environmental protection and health standards. Such a system can minimize health disparities and promote the population’s general health.

#### 2.1.1 Economy

The regulation and development of the economy by the political-economic system directly impact on the population’s health. The stability and development of the political-economic system directly influence a country’s overall economic situation. This, in turn, affects people’s income, employment opportunities, living conditions, investment, and the development of healthcare facilities. Consequently, these factors impact individuals’ health and lifestyles (13). Economic prosperity is typically associated with improving people’s living standards, enabling them to purchase quality food and improved living conditions. This, in turn, can positively impact their nutritional status and lifestyle habits. Furthermore, economic prosperity can facilitate the provision of superior medical facilities, which can enhance living standards and promote improved nutritional conditions, thereby contributing to an overall improvement in public health. Conversely, economic depression can result in a decline in the standard of living, an increase in food insecurity, and a corresponding increase in malnutrition and health problems (14). Furthermore, economic poverty can result in a dearth of healthcare resources, heightened disease transmission, and an increased prevalence of health issues (15). To illustrate, following the financial crisis of 2009, the prevalence of chronic obstructive pulmonary disease (COPD) in Greece increased to 18.2%, a notable rise from the 5.6% prevalence observed prior to the crisis. This surge can be attributed to various factors, including declining wages, rising unemployment, reduced pensions, and diminished treatment adherence (16, 17). The stability and development of the political-economic system directly influence employment opportunities and living conditions. In periods of economic growth, employment opportunities expand, enabling individuals to secure stable incomes and enhanced living standards. This, in turn, mitigates the adverse effects of poverty and unemployment on health outcomes. Economic downturns and poverty can precipitate an increase in unemployment and a deterioration in living conditions, which, in turn, can have a deleterious impact on people’s health. For example, during the COVID-19 epidemic, a significant number of workers were rendered unemployed as a consequence of the government’s closure and control measures. This resulted in a decline in their socio-economic status. At the same time, the people’s standard of living decreased due to the lack of essential goods and services, which led to various subsequent health problems (18).

Furthermore, the state of the political-economic system directly impacts the investment and development of healthcare facilities and resources at the national level. In periods of economic growth, the government and private sector typically increase investment in healthcare facilities and enhance the quality and scope of healthcare services, thereby facilitating more timely and effective access to healthcare for the population. Conversely, during periods of economic downturn and poverty, healthcare resources may be constrained, leading to insufficient healthcare services and a deterioration in quality, which has a detrimental impact on public health. For example, the United Kingdom’s economic recession has resulted in a notable decline in the financial resources allocated to healthcare. This has led to an increase in the prevalence of conditions such as disability, cancer, and cardiovascular diseases, contributing to a lower performance in terms of premature mortality compared to other European Union (EU) countries (19, 20). Conversely, as China’s economy expands, its regionalization has made noteworthy advancements. A 1% increase in regional integration has resulted in improvements of 6.6% and 1.9% in the healthcare workforce and healthcare infrastructure, respectively. These improvements contribute to enhanced public health and wellbeing (21).

#### 2.1.2 Welfare

The social welfare policies embedded within the political-economic system, including public service and infrastructure, healthcare system, and education, directly influence population’s health. First, the political-economic system influences the extent of public services and the advancement of infrastructure. Some studies have indicated an imbalance in infrastructure and public services between urban and rural areas in developing countries. This imbalance is more pronounced in urban areas than in rural areas, which has a significant impact on public health (22, 23). In response, China has proposed the ‘Broadband China Strategy and Implementation Plan’, which aims to enhance the quality of life for its citizens by improving digital infrastructure. This initiative is expected to result in significant cost savings for residents, with an estimated reduction of up to 18.8% in healthcare expenditures (24). A country’s political- and economic system also influences the development of its social security system, which encompasses unemployment insurance, pensions, and disability benefits. A well-developed and sound social security system alleviates the burden on individuals, increases the probability of citizens having access to timely medical care and necessary livelihood security, enhances their quality of life, thereby reducing health problems caused by living in straitened circumstances, and contributes to the maintenance of physical and mental health. To illustrate, in 2016 the United Kingdom All-Party Parliamentary Group on Health in All Policies published the findings of the ‘Welfare Reform and Work Bill 2015-2016 on the health of children living in poverty (25). The legislation imposes limitations on the benefit cap and freezes benefits for a 4-year period, impacting the income levels of thousands of impoverished families. This consequently gives rise to an elevated prevalence of childhood poverty and a greater incidence of cognitive, emotional, and behavioral disorders, which in turn give rise to a range of health issues, including asthma, obesity, smoking, early pregnancy, and self-harm.

Moreover, a country’s political-economic system serves to shape its healthcare system. The provision of universal healthcare coverage represents a pivotal determinant of the health status of a country’s population (26). In socialist countries, the government provides a public healthcare system, whereby all citizens can access healthcare services free of charge or at a nominal cost. In capitalist countries, the healthcare system is more market-oriented, and individuals must purchase health insurance or pay out-of-pocket to access healthcare services. This impacts the manner, quality, and cost of access to healthcare services, which in turn affects the level of health attained by individuals. A policy of universal healthcare coverage can ensure that all individuals have access to the requisite healthcare services, thereby enhancing overall health outcomes. Indonesia being the fourth most populous country in the world, the government of Indonesia has been working to achieve universal healthcare coverage for people with low incomes in remote areas. This has resulted in a significant decline in neonatal and child mortality rates. The infant mortality rate decreased from 67.8/1,000 in 1992 to 34.0/1,000 in 2007, and the mortality rate for children under 5 years of age declined from 97.4/1,000 to 45.0/1,000 (27).

Ultimately, the level of education is closely correlated with the level of health, and the educational policies enacted by political-economic systems directly influence the level of education attained by the population. Individuals with higher levels of education tend to have more comprehensive access to health-related knowledge, engage in healthier behaviors, and are more likely to secure well-paid jobs and stable employment, which in turn contributes to their overall health and wellbeing (28, 29). For example, it is estimated that in the 40 years between 1970 and 2009, the global number of deaths of children below 5 years of age fell by 8.2 million, with half of this decline attributed to improvements in women’s educational attainment (29).

#### 2.1.3 Environment

Economic development represents a significant avenue for enhancing living standards, curbing poverty, and ensuring the provision of fundamental public services. However, the pursuit of economic development frequently results in the depletion of natural resources and the contamination of the environment. The considerable energy consumption and waste emissions associated with industrialization and urbanization can potentially exert significant adverse effects on the environment. Consequently, the challenge of balancing economic development and environmental protection represents a pivotal issue in formulating political-economic policies. The policies established and executed by the political-economic system directly influence the quality of the living environment, and, thus, the population’s health. The prevalence of industrialization and environmental contamination in a given area is associated with an elevated risk of illness among the population. Conversely, implementing robust environmental protection measures and establishing rigorous health standards can effectively mitigate this risk (30). The objective of sustainable development policies is to safeguard the natural environment and guarantee the sustainable utilization of resources throughout the process of economic growth. The government can facilitate the transition of the economy to a green, low-carbon, and circular economy in two principal ways: through the enactment of the legislation, the implementation of economic incentives, and the advancement of technological innovation; and through the establishment of environmental taxation and subsidy policies, which provide incentives for enterprises and individuals to adopt environmentally friendly behaviors. Such measures include imposing taxes on pollution emissions and the provision of subsidies to businesses that utilize clean energy and environmentally friendly technologies. Furthermore, a country’s political-economic system exerts an influence on implementing its environmental policies, including the regulation of pollution and the management of natural resources (31, 32). The degree to which the government prioritizes environmental protection establishes rigorous environmental standards and enhances pollution control directly impacts the quality of the living environment. Some countries have implemented environmental regulations and policies to enhance environmental quality by regulating pollution and safeguarding natural resources, thereby reducing the likelihood of adverse health outcomes. A country’s political-economic system also exerts an influence on the management of its natural resources (33). Implementing policies about energy development, land use, and water management can exert a considerable influence on the quality of the natural environment. Implementing reasonable natural resource management strategies can mitigate environmental degradation and contamination, sustain ecological equilibrium, and enhance human wellbeing. However, formulating environmental policies frequently entails the participation of many stakeholders, including government departments, private enterprises, non-governmental organizations, and the general public. The needs and interests of these stakeholders may conflict with one another, and policymakers must reconcile the various interests. To illustrate, the government of El Salvador rejected the Australian–Canadian mining company Oceana Gold’s proposal to extract gold from the country’s soil. This decision was based on the environmental concerns surrounding the potential pollution of water resources resulting from gold exploration and mining activities. Consequently, Oceana Gold initiated legal proceedings against El Salvador, seeking damages of millions of dollars (34). In the context of globalization, the political-economic system also influences international cooperation and environmental protection (35). The development and implementation of environmental policies at the international level frequently necessitates the collaboration of multiple countries. However, discrepancies in countries’ environmental policies may result in a phenomenon known as ‘free-riding’ in environmental protection. International environmental agreements and cooperation mechanisms, such as the Paris Agreement, are designed to facilitate the coordination of national environmental policies and to enable collective action to reduce CO₂ emissions to address global environmental challenges (35). Indeed, reducing greenhouse gas emissions confers benefits not only to cardiopulmonary health but also to the economy, thereby creating a positive feedback loop (36, 37). The degree to which the political-economic systems engage with international environmental cooperation directly impacts the efficacy of global environmental protection, which in turn influences the health and wellbeing of people (38).

Environmental policies in a market economy are primarily based on market mechanisms. For example, implementing carbon trading markets and green finance incentivizes enterprises to reduce carbon emissions and invest in environmentally friendly projects. Nevertheless, market failures, such as externalities, necessitate governmental regulation and intervention. In a planned economic system, the government is able to exercise direct control over the allocation of resources and the activities of producers and formulate and implement policies designed to protect the environment rigorously. Nevertheless, a planned economic system may encounter information asymmetry and inefficiency challenges. A mixed economic system is a hybrid of market and planned economies that aims to advance environmental protection through the dual roles of the government and the market. The government establishes environmental regulations and standards, while the market mechanism provides innovation and efficiency, thus complementing each other. The European Union has a policy system for environmental protection that is unparalleled on a global scale. This system includes stringent emission standards, carbon trading markets, and renewable energy development targets (39, 40). These policies have significantly improved economic green transformation, curbing pollution, and combating climate change. China is currently experiencing a significant environmental crisis, particularly given the rapid pace of economic growth. In recent years, the Chinese government has proposed the concept of ‘green water and cyan mountains are golden and silver mountains’, which advocates for the integration of environmental protection and economic transformation through the implementation of rigorous environmental legislation, extensive afforestation initiatives, and investments in clean energy (41). The fluctuations in the political landscape significantly shape the environmental policy of the United States. There are notable discrepancies in environmental regulations, energy policies, and climate change stances across different presidential administrations. To illustrate, numerous environmental regulations were eased during the Trump administration, whereas the Biden administration re-engaged with the Paris Agreement and proposed a comprehensive green infrastructure initiative (42, 43).

The relationship between the environment and human health is complex, with numerous factors influencing the health status of individuals. These include air quality, water quality, soil pollution, noise pollution, chemical pollution, and climate change. Air pollution represents a significant public health concern on a global scale. In 2015, an estimated 9 million premature deaths were attributed to pollution, representing 16% of all global deaths. This figure is three times higher than the number of deaths associated with acquired immunodeficiency syndrome (AIDS), tuberculosis, and malaria combined and 15 times higher than the number of deaths from all wars and other forms of violence (44). The impact of environmental pollution on human health is not uniform across different regions. For instance, in sub-Saharan Africa, environmental pollution represents the primary cause of disease (45). Airborne pollutants, including particulate matter (e.g., PM2.5 and PM10), nitrogen dioxide (NO_2_), sulfur dioxide (SO_2_), and ozone (O_3_), have been demonstrated to cause significant adverse effects on multiple organ systems, particularly the respiratory, cardiovascular, and central nervous systems (46). Long-term exposure to elevated concentrations of PM has been linked to an increased prevalence of chronic bronchitis, asthma, lung cancer, hypertension, atherosclerosis, heart disease, stroke, diabetes, obesity, Alzheimer’s disease, Parkinson’s disease, and other adverse health outcomes (45–49). Another significant environmental health concern is water pollution. Deleterious chemicals, heavy metals, and pathogenic microorganisms in polluted water sources have been linked to various adverse health outcomes. Ingestion of contaminated water sources has been linked to the development of gastrointestinal diseases, including diarrhea, cholera, and dysentery (50). The long-term consumption of water sources contaminated with heavy metals (e.g., lead, mercury, and arsenic) has been linked to an increased risk of developing chronic diseases, including cardiovascular disease, kidney disease, liver disease, diabetes, neurological damage, and cancer (51). A study revealed that a total of 5% of disability-adjusted life-years (DALYs) in 38 countries were attributable to water contamination. In particular, the proportion was as high as 16% in Chad (45). The contamination of soil can have a detrimental impact on the safety of crops and drinking water sources. The presence of contaminants in soil may result in the leaching of these substances into groundwater, which can subsequently impact the quality of drinking water sources. The ingestion of crops cultivated in contaminated soil may result in the absorption of heavy metals, pesticides, and other toxic substances through the food chain, potentially leading to adverse health outcomes. These include chronic hypertension, kidney failure, cardiovascular disease, stroke, poisoning, cancer, and neurological developmental defects in children (44). Additionally, noise pollution represents a significant concern in urban settings. Long-term exposure to high-intensity noise has been linked to a range of adverse health outcomes, including noise-induced hearing loss, as well as increased stress, hypertension, heart disease, poor sleep quality, cognitive impairment, anxiety, and depression (52–54). The detrimental effects of chemical pollution on human health can be attributed to many factors, including industrial emissions, wastewater, pesticides, household chemicals, and other anthropogenic sources. Acute toxic symptoms, including headache, nausea, vomiting, and respiratory distress, may result from short-term exposure to high concentrations of toxic chemicals. Conversely, chronic diseases such as cancer, heart disease, COPD, asthma, endocrine disorders, neurodegeneration, and reproductive health problems may result from long-term exposure to low concentrations of toxic chemicals (55–58). Extreme weather events, which are a consequence of climate change, such as high temperatures, heavy precipitation, and droughts, directly impact human health. Furthermore, these events may also result in the development of mental health issues, including post-traumatic stress disorder (PTSD) (59). Elevated temperatures may precipitate a range of health concerns, including heat stroke, heat exhaustion, and the exacerbation of cardiovascular disease. Conversely, they may also influence the distribution of vector insects (e.g., mosquitoes and ticks) and enhance the risk of infectious disease transmission, such as dengue fever, malaria, and Lyme disease (60, 61). Furthermore, climate change may also impact food production and supply, potentially leading to food shortages and malnutrition issues. For example, the Saami people living in Norway, Sweden, Finland, and the northern part of the Kola Peninsula in Russia depend on reindeer herding, fishing, and hunting for their livelihoods. However, global warming has reduced the distribution of lichen, the herd’s main food source, which has had a detrimental impact on the Saami people’s food security and cultural practices, leading to an increase in disease prevalence (62).

### 2.2 Regulatory system

Regulatory systems exert a significant influence on human health. Regulatory systems are comprised of rules, standards, and policies developed and enforced by governments or other agencies to protect the public interest, maintain social order, and promote public safety. The regulatory system plays an instrumental role in ensuring food and drug safety, regulating pollutant emissions, safeguarding occupational safety, guaranteeing the quality of medical services, and protecting consumer rights. These functions collectively impact public health. A robust regulatory system serves not only to prevent and control health risks effectively but also to promote health equity and enhance society’s overall health. Therefore, it is evident that enhancements to and fortification of the regulatory system in the health sector are of paramount importance in the protection and advancement of public health.

#### 2.2.1 Food and drug security

The regulatory system is responsible for monitoring and managing the quality, safety, and efficacy of food and drugs, thereby playing a pivotal role in ensuring food and drug safety (63). The safety of food products is directly correlated with human health. There is a growing body of evidence indicating that the consumption of unsafe food may contribute to an increased risk of developing cardiovascular disease (64, 65). The government employs a multifaceted approach to prevent food contamination and foodborne illnesses. This is achieved through the enactment and enforcement of comprehensive food safety regulations. These regulations ensure that food products meet the requisite safety standards at all stages of the production, processing, transport, and distribution cycle. The responsibility of food regulatory agencies is to approve, monitor, and inspect food production, processing, and distribution processes. They ensure that food producers comply with health and safety standards, that food products are free of hazardous substances, and that labeling instructions are followed. The regular inspection of food manufacturers, the regulation of food additives, and the monitoring of food labeling and packaging are all integral components of food safety regulation. By implementing rigorous supervision and regulation of the production, processing, sales, and utilization processes, consumers can procure food with the assurance of its safety and quality, thereby averting potential health hazards such as foodborne illness and adverse drug interactions, and promoting public health and safety (66). In the United States, 12% of outbreaks and 20% of outbreak-related diseases are attributable to food insecurity. This results in 48 million cases of illness and 3,000 deaths annually, with direct and indirect economic losses reaching 1.4 trillion dollars (67). In response, the United States government introduced the Food Safety Modernization Act, which aims to regulate the production, transportation, packaging, and processing of food to reduce foodborne illness (67). Consequently, by formulating and enforcing pertinent legislation and regulations and establishing corresponding regulatory agencies, such as the Food and Drug Administration, the government can oversee the production, processing, distribution, and utilization of food and pharmaceuticals to guarantee their adherence to established standards and requirements. This is performed to prevent the occurrence of potential health risks, such as foodborne illness and adverse drug reactions, and to protect public health (68, 69).

On the one hand, regulatory agencies conduct regular inspections and supervision of the production, processing, distribution, and use of food and drugs. The objective of these inspections is to ensure that enterprises and individuals comply with the relevant laws and regulations and to guarantee the quality and safety of the products. On the other hand, these agencies can disseminate information and provide education to the public regarding food and drug safety, thereby enhancing the public’s knowledge and awareness of food and drug safety and facilitating informed decision-making (70, 71). For example, in 2004, the United States Food and Drug Administration (FDA) required that manufacturers of antidepressant medications include a warning on the labels of their products regarding the potential risk of suicide in pediatric populations and the necessity of closely monitoring patients taking these medications. Subsequently, there was a notable decline in the proportion of pediatric patients diagnosed with depression who were taking these medications (72). Implementing these measures enables the regulatory system to effectively prevent health risks, such as food poisoning and medication side effects, and safeguard the population’s health and safety.

#### 2.2.2 Pollutant emission

In the contemporary era, the regulatory apparatus has expanded its purview to encompass not only the monitoring of food and drug quality but also the formulation of policies and the establishment of standards about environmental protection. These include the regulating pollutant emissions and managing waste disposal, to maintain environmental quality. Clean air, water, and soil are indispensable to human health, and effective environmental regulation can mitigate the health risks associated with environmental pollution. Primarily, the regulatory system guarantees that industries adhere to environmental regulations and curtail the discharge of pollutants throughout the production and operational phases by establishing rigorous environmental protection policies and standards. Such policies and standards can regulate the behavior of enterprises and motivate them to adopt environmental protection measures to reduce the negative impacts on the environment (73). The ‘Two Control Zones’ policy represents the earliest and most rigorous environmental policy in China, aiming to regulate the emission of SO₂ from factories and enterprises, controlling acid rain, and enhancing air quality. Since its implementation in 1998, there has been a 5.7% decline in respiratory morbidity, with more significant public health impacts observed in central and western cities and provincial capitals (73).

Furthermore, the regulatory system oversees and regulates pollutant emissions to safeguard air quality and mitigate the adverse effects of air pollutants on the human respiratory system. This is achieved by monitoring and limiting emissions from industrial facilities, vehicles, and other sources of pollution. As an illustration, China’s regulation of fireworks displays has resulted in a reduction of PM2.5 emissions and an alleviation of air pollution, which has led to enhanced public health and a decline in the prevalence of respiratory and cardiovascular diseases (74). Furthermore, regulatory bodies have encouraged the implementation of clean energy and emissions reduction technologies to reduce carbon emissions and other forms of pollutant emissions (75). As evidenced by statistical data from 2020, clean energy sources account for the majority of residential energy consumption in several countries. In the United States, for instance, they represent 94.7% of the total; in Germany, the figure stands at 86.8%. In China, the proportion is 57.5%, and in India, it is 35.7%. South Africa, on the contrary, has a slightly lower rate of 32.1% (76). The recently unveiled ‘Clean Energy Strategy’, a policy initiative spearheaded by the Chinese government, seeks to bolster the utilization of clean energy sources and curtail air pollution. China’s subsidies and regulatory framework for clean energy heating in the northern regions have reduced winter-related healthcare costs, including expenditures on medicines, healthcare products, medical devices, healthcare equipment, outpatient care, and hospitalizations. This reduction in costs has offset the additional costs associated with subsidizing clean energy, suggesting that promoting clean energy not only enhances public health but also generates economic benefits (77).

Moreover, the regulatory system bears is responsible for overseeing waste disposal practices, ensuring that wastes are handled and disposed of appropriately and that they do not contaminate soil and water sources. By establishing waste treatment standards and monitoring the operation of waste treatment facilities, regulatory agencies can effectively mitigate the adverse effects of waste on the environment and human health. For example, the accelerated proliferation of electronic products in the 21st century has generated a considerable quantity of e-waste, estimated at up to 72 million tons. This e-waste can contribute to the proliferation of plastic pollution and toxic e-waste (78). This e-waste has attracted significant attention from the international community, establishing the Basel Convention on the Control of Transboundary Movements of Hazardous Wastes and their Disposal and the Waste of Electrical and Electronic Equipment Directive. These instruments mandate that producers, sellers, and consumers share the financial burden of recycling and disposal, to reduce pollution (79, 80).

#### 2.2.3 Occupational safety

The regulatory system is designed to guarantee workplace safety, and it encourages departments to implement safety training, provide safety facilities and protective equipment, and collect health data to enhance protective measures. Primarily, the health of employees can be safeguarded through the enactment of legislation, the observation of occupational settings, and the implementation of safety training programs (81). Regulators are responsible for overseeing the work environment and ensuring that it adheres to the requisite safety and health standards. This entails conducting periodic inspections and assessments of the workplace, to identify potential hazards and risk factors. The prompt identification and mitigation of safety hazards in the workplace is an effective strategy for reducing the risk of occupational injuries and illnesses (82). The Occupational Safety and Health Act establishes minimum safety and health standards for workplaces across all sectors. It also conducts regular medical examinations of workers exposed to toxic substances and monitors employers’ compliance with safety regulations and the results of workplace inspections. The goal of these measures is to ensure the public’s health and safety and to reduce workplace fatalities (83). It is frequently the case that regulatory agencies mandate that employers provide their employees with the requisite safety training to ensure that employees are able to identify and respond to potential hazards in the workplace. Such training may encompass the proper utilization of tools and equipment, the procedures for responding to emergencies, and the methods for preventing occupational diseases. The provision of comprehensive safety training enables employees to safeguard the health of workers more effectively (84). A team of researchers in the United States studied safety training for the logging industry. The study encompassed a range of topics, including injuries and fatalities associated with logging, safety conditions, tragic events in the industry, resolution of real-world scenarios, hazard identification and control, and timely reporting and resolution of safety hazards. The study revealed that following the training, there was a notable increase in the frequency of safety discussions among workers and a corresponding rise in the proportion of daily safety inspections. These findings indicate that safety training plays a pivotal role in enhancing workers’ safety awareness and safeguarding their wellbeing (85).

To guarantee the safety of employees at work, regulators have issued a recommendation that employers provide the necessary protective facilities and equipment. Such provisions may include the provision of personal protective equipment (e.g., helmets, goggles, earplugs, etc.), the establishment of emergency facilities (e.g., fire extinguishers, first aid kits, etc.), and the implementation of effective design principles for the work environment (e.g., ventilation systems, non-slip flooring, etc.) (86). For example, in New Zealand, where skin cancer is a prevalent and costly health concern, the provision of sun protection equipment, such as sun hats and sun creams, for workers engaged in outdoor activities during the summer months can effectively mitigate the adverse effects of ultraviolet radiation on the skin, including sunburn. It may potentially reduce the risk of developing skin cancer (87). It is incumbent upon regulators to emphasize the importance of the proper use and maintenance of these facilities by employers, to minimize the risk of work-related injuries and occupational diseases (88).

Furthermore, regulators are able to collect and analyze data regarding workplace safety to evaluate the efficacy of existing standards and identify potential improvement areas. By employing data-driven decision-making, regulators can enhance their comprehension of the potential hazards in the workplace. This may entail the utilization of job safety analysis or safety data sheets to evaluate the risks that may be present and implement measures to safeguard the wellbeing of employees (89). A data-based study revealed that extended shifts and limited rest periods result in diminished productivity and increased worker safety risks. The implementation and enhancement of shift work systems not only safeguard employee safety but also enhance productivity (90). Throughout the process, regulators collaborate with employers, employees, and other stakeholders to ensure the safety and health of the workforce (86).

#### 2.2.4 Quality of medical service

The regulatory system is responsible for monitoring the conduct of healthcare facilities and healthcare professionals, to guarantee the quality and safety of healthcare services. This encompasses licensing healthcare facilities, medical practitioners, and the approval of pharmaceuticals and medical devices. Additionally, it includes the quality control of healthcare services and the protection of patients’ rights and safety. Providing high-quality healthcare services is a fundamental aspect of maintaining public health and wellbeing (91). The regulatory system serves two distinct yet complementary functions. First, it ensures that healthcare organizations obtain the requisite permission from the relevant authorities before commencing operations. This is performed to guarantee that the facilities in question comply with the relevant health standards and safety regulations. Second, the regulatory system allows for regular inspections and audits to be conducted. This is performed to ensure that the healthcare facilities continue to meet the required standards. The regulatory system may oversee the administration of national or regional licensing examinations to medical professionals, to ensure the safety of the medical profession. Implementing a registration and recertification system for physicians and nurses guarantees the maintenance of their licenses to practice. Furthermore, the establishment of continuous education and training requirements ensures that healthcare professionals continuously update and enhance their professional knowledge and skills, thereby improving healthcare quality. For example, under the supervision of the National Institutes of Health, the Cardiovascular Institute at Stanford University offers a variety of training programs, including postdoctoral training, early career mentorship programs, high school, premed, and undergraduate summer research programs, among others. These programs expose trainers to the latest cardiovascular imaging research techniques and mechanisms of vascular biology and myocardial biology, thus enhancing theory and practice and improving medical therapy (92). The sale of drugs and medical devices on the market is contingent upon their successful completion of a rigorous clinical trial and approval process, which aims to guarantee their safety and efficacy. The utilization of marketed drugs and devices is subject to regular monitoring and evaluation, thereby facilitating the identification and timely resolution of potential safety concerns. For example, the European Medicines Agency’s Pediatric Committee, under the supervision of the European Union, adopted the Pediatric Regulation, which requires pharmaceutical companies to monitor and investigate the indications and adverse reactions of pediatric medicines and improve drug safety (93). The regulatory system establishes standard operating procedures and clinical guidelines to standardize the provision of healthcare services (94). For example, the 2016 classification of central nervous system (CNS) tumors, which defines and classifies CNS tumors based on histological features of the tumor, was developed under the guidance of the World Health Organization (WHO). The new version of the 2021 guidelines, which defines and classifies CNS tumors based mainly on molecular genetic features, introduces novel concepts to the classification, diagnosis, and treatment of CNS tumors (95, 96). Furthermore, implementing quality management systems and high-reliability organizations can enhance healthcare organizations’ managerial capacity and service quality (97). Early quality improvement in healthcare can be traced back to the 19th century, when Ignaz Semmelweis, an obstetrician, incorporated handwashing into healthcare practices. The necessity of surgical handwashing before surgical procedures directly results from his pioneering work (97). Initiatives to enhance the quality and dependability of care are now being implemented in select locations. One such initiative is the New York State Cardiac Surgery Reporting System, which optimizes cardiovascular surgery by aggregating hospital clinical data (98). A framework of laws and regulations exists to ensure that patients are able to access information and exercise choice. Establishing patient complaint and suggestion channels enables the prompt response and resolution of patient concerns and problems. Promoting a patient safety culture encourages healthcare organizations and personnel to priorities patient safety and take the initiative to implement improvements. The collation and examination of medical incidents and patient grievances can facilitate the formulation of corrective measures to avert the reoccurrence of analogous incidents (99). For example, a study by the National Health Service (NHS) indicated that organizations subject to complaints should be aligned with the complainant from the outset. Furthermore, handling complaints can be enhanced through linguistic analysis and reference to the consumer literature’s justice-based approach (100).

#### 2.2.5 Consumer right

The regulatory system is of paramount importance in safeguarding consumer rights, which encompasses product safety regulation and service standards guarantee. A robust regulatory system is a vital component in maintaining public health and the general wellbeing of society. The regulatory system safeguards consumers’ rights and interests, deterring fraud, false advertising, and mis-selling practices. It also ensures that the goods and services purchased by consumers comply with the relevant standards and protects the public from all kinds of health threats by establishing rules, monitoring enforcement, and imposing penalties when necessary. Primarily, the regulatory system is concerned with the quality and safety of products (101, 102). In the context of manufacturing and other product-related sectors, regulatory agencies are responsible for approving and monitoring the process of product design, production, and distribution. They are responsible for ensuring that products comply with the relevant standards and regulations and do not cause harm or damage to consumers. For instance, electronic products are required to comply with established electrical safety standards. At the same time, toys must adhere to specified child safety standards to prevent excessive levels of hazardous substances. The United States Environmental Protection Agency (EPA) maintains a list of over 40,000 currently registered chemical substances (103). In 2019, the state of Washington passed the ‘Pollution Prevention for Healthy People and Puget Sound Act’, which aims to reduce the levels of toxic chemicals in consumer products, including phthalates, per- and polyfluoroalkyl substances (PFAS), phenolic compounds, flame retardants, and polychlorinated biphenyls (PCBs). This legislation enhances product safety and protects public health (104). Establishing and enforcing regulatory standards enhance product quality and mitigate the risk of consumers being exposed to substandard or hazardous products.

Furthermore, the regulatory system guarantees the maintenance of service standards. Similarly, service industries such as education and finance must be subject to monitoring and regulation by the relevant regulatory bodies (105–107). They guarantee that service providers comply with industry norms and standards, ensuring high-quality, reliable, and safe services. The responsibility for ensuring equity and quality in education lies with government regulators, who must guarantee that all individuals can access educational resources. Consequently, the quality of education and citizens’ health are inextricably linked. Individuals with higher levels of education are more likely to pursue lucrative careers, adopt salutary lifestyles, and possess a more profound understanding of healthcare, which collectively enhances their wellbeing (29). To illustrate, in June 1999, the Central Committee of the Communist Party of China and the State Council convened the third national conference on education since the Reform and Opening Up period. The ‘Decision on Deepening Educational Reform and Comprehensively Promoting Quality Education’ was unveiled at this conference. The conference exhorted the entire Communist Party and the people of all nationalities to become more proactive in enhancing national civilization and innovation, fortifying the institutional and structural reform of the education system, and comprehensively advancing quality education, to revitalize the education industry as part of the national strategy of developing the country through science and education. Over the past two decades, China’s educational policy has espoused a philosophy of universal education, with many reforms implemented across various sectors. These reforms have significantly enhanced the quality of education for the entire population (107).

### 2.3 Policy

The relationship between policies and people’s health is a complex one. The formulation and implementation of policies have far-reaching impacts on people’s health conditions, mainly related to healthcare and public health policies. By employing scientific and reasonable policy formulation and effective implementation, the government can markedly enhance the public’s health status, diminish health inequality, and elevate the collective health of society. Therefore, it is evident that the research and optimization of health policies are of great significance to the realization of health for all.

#### 2.3.1 Public health policy

Public health policies are defined as those formulated and implemented by the government to safeguard the population’s health. Such policies encompass a range of areas, including disease prevention, health promotion, control of infectious diseases, and improvement of the quality of medical services. By way of illustration, vaccination programs, anti-smoking legislation, control of contagious diseases, and so forth represent components of public health policies that directly influence the health of the general population. Vaccination programs represent a crucial element of public health policies, safeguarding populations from infectious diseases through implementing vaccination strategies (108). Implementing mass vaccination programs has been demonstrated to be an effective strategy for the control and, in some cases, the eradication of serious infectious diseases, including smallpox, measles, and polio (109, 110). The implementation of vaccination programs by governmental bodies serves to safeguard the health of not only the individual vaccinator but also the collective health of the population, thereby establishing a state of herd immunity.

Smoking addiction represents a significant global public health concern, with 1 billion men and 250 million women engaging in tobacco use worldwide, according to data from the WHO (111). The consumption of tobacco products is a significant risk factor for the development of numerous chronic diseases, including lung cancer, cardiovascular disease, and COPD (112–115). To mitigate the public health risks associated with smoking, numerous countries and regions have enacted anti-smoking legislation. Anti-smoking legislation encompasses a comprehensive array of measures, including a complete prohibition on smoking in public spaces and workplaces, limitations on tobacco advertising and promotion, the mandatory display of health warning images on cigarette packaging, and increased taxes on tobacco products. These measures contribute to a reduction in smoking prevalence and the adverse effects of passive smoking on non-smokers while also enhancing overall public health.

The public health policy encompasses a range of control measures designed to prevent the spread of infectious diseases. These include disease surveillance, quarantine and isolation, the notification of outbreaks, and the dissemination of public health education. Implementing a comprehensive infectious disease surveillance system enables the government to promptly identify and respond to disease outbreaks, implement effective measures to control the spread of epidemics, and safeguard public health. For example, during the COVID-19 pandemic, governments implemented a series of measures, including travel restrictions, social distancing, and the use of masks, to control the spread of the virus and protect public health (116).

Health promotion policies aim to encourage and assist individuals in adopting healthy lifestyles, preventing the onset of chronic diseases, and enhancing the quality of life. The government may promote healthy diets, physical exercise, and mental health through public health promotion, community health programs, and school health education. For example, communities are encouraged to organize fitness activities, provide guidance on healthy diets, and set up mental health counseling services. In response to the COVID-19 pandemic, the government employed a multifaceted approach to mitigate the impact of the virus. This included issuing risk warnings, influencing individual behavior, disseminating information about symptoms, and providing guidance on medication (117).

#### 2.3.2 Healthcare policy

The field of healthcare policy encompasses a range of crucial aspects, including the distribution of healthcare resources, the structure and delivery of healthcare services, and the development of a robust medical insurance system. The government’s healthcare policy significantly impacts how individuals access healthcare services, the quality and cost of those services, and consequently, their overall health status. The distribution of healthcare resources represents a fundamental aspect of healthcare policy. It is incumbent upon the government to allocate medical facilities, equipment, capital, and human resources rationally, to ensure that different regions and different groups of people have access to necessary medical services. The equitable and efficient allocation of healthcare resources, as exemplified by the Affordable Care Act in the United States, can reduce health inequalities and enhance the accessibility of healthcare services (118). For instance, in rural and remote regions where resources are scarce, the government can enhance the distribution of healthcare resources by constructing primary healthcare facilities, providing mobile healthcare services, and training and deploying healthcare professionals. The mode of delivery of healthcare services is also a crucial aspect of healthcare policy and encompasses diverse forms, including public hospitals, private hospitals, and community health service centers. By implementing policy regulations and providing support, the government can facilitate the coordinated development of public and private healthcare institutions, thereby ensuring the quality and accessibility of healthcare services. Furthermore, the government can facilitate the implementation of novel medical services models, such as family doctor systems, telemedicine services, and short-term medical services, to address the heterogeneous medical requirements, particularly in remote and resource-scarce regions (119–121).

The medical insurance system serves as a crucial mechanism for ensuring that individuals have the financial means to access necessary medical care (122). The government’s establishment and enhancement of the medical insurance system provides individuals and families with a fundamental level of medical protection, while simultaneously alleviating the financial burden associated with healthcare (123). The medical insurance system encompasses a range of forms, including universal health insurance, employee health insurance, resident health insurance, and other variants, collectively providing coverage to diverse groups of individuals. An optimal health insurance system can enhance the accessibility of healthcare services, facilitate the improvement of healthcare service quality, and promote the rational use of healthcare resources by regulating health insurance payment mechanisms. The government’s healthcare policy directly influences the quality and cost of healthcare services. To enhance the quality of healthcare services, the government can establish and enforce healthcare service standards and norms, conduct healthcare quality monitoring and assessment, and encourage the advancement of healthcare technology and medical research. Furthermore, the government can regulate medical expenses, prevent excessive growth in medical costs, and reduce the financial burden on patients through measures such as the implementation of a health insurance payment system, the regulation of drug prices, and the pricing of medical services (124). The implementation of rational healthcare policies enables the government to markedly enhance the population’s health. For example, the promotion of essential public health services, the implementation of significant disease prevention and control programs, and the strengthening of chronic disease management can effectively reduce the incidence of disease and mortality rates, thus improving the health of the population (125). Furthermore, the government can facilitate the adoption of healthy behaviors and the prevention of disease through the implementation of health education and health promotion policies (126).

### 2.4 Commercial entity

Commercial entities have a significant impact on health, both beneficial and detrimental. On the one hand, commercial entities positively influence public health through activities such as sponsoring scientific research, providing medical services, producing medicines and medical devices, and promoting healthy lifestyles. On the other hand, their behavior and motivations can also lead to negative outcomes, including health risks and inequalities. By employing innovative strategies and market-driven approaches, commercial entities can provide the public with high-quality medical services, medicines, and health products while promoting healthy lifestyles. However, the profit motive of commercialized operations may also result in adverse health outcomes, including elevated costs, disparities in service accessibility, and deceptive marketing practices. It is thus incumbent upon the government and society at large to ensure that the actions of commercial entities are conducive to public health, through the implementation of effective regulatory and supervisory measures, and to promote a balance between commercial interests and social health objectives.

#### 2.4.1 Scientific research

Many pharmaceutical companies and medical technology businesses are engaged in the research and development of novel drugs and medical technologies with the potential to facilitate the creation of new treatments, medicines, and medical devices for a diverse array of diseases, thereby enhancing public health. For example, the advent of new drugs for cancer, cardiovascular diseases, and autoimmune diseases, in addition to advanced medical devices and surgical techniques, has the potential to markedly enhance patient’s survival rate and quality of life. Vaccines represent an efficacious method of preventing infectious diseases, and commercial entities play a pivotal role in vaccine research and production. By conducting comprehensive research on disease pathogens and immunological mechanisms, commercial entities can develop safer and more efficacious vaccines for controlling the spread and prevalence of diseases. In particular, during outbreaks of infectious diseases, the vaccine research and production capacity of commercial entities is of paramount importance for the protection of public health and safety. To illustrate, in response to the global pandemic of COVID-19, BioNTech and Pfizer developed the BNT162b2 vaccine, a lipid nanoparticle-formulated, nucleoside-modified RNA vaccine that provides 95% protection against COVID-19. This vaccine has been instrumental in preventing the spread of the virus (127). By employing big data analytics and health information technology research, commercial entities can leverage the potential value of health data to identify disease prevalence trends, risk factors, and preventive measures. The findings of such research can assist healthcare organizations and governments in developing more efficacious health policies, preventive measures, and public health promotion activities, to prevent and control the spread of diseases. To illustrate, the eICU-Collaborative Research Database, developed under the auspices of Philips Healthcare, encompasses medical data from 208 hospitals in the United States on over 200,000 cases. This data includes patient vital sign measurements, care plan documentation, disease severity measurements, diagnostic information, and treatment information. These data can be utilized to enhance the management of critically ill patients and to inform policy development in the health sector (128). The research conducted by commercial entities in the food and nutraceutical industries regarding nutrition and health products has the potential to result in the development of dietary choices and nutritional supplements that can contribute to improving people’s dietary profiles and lifestyles. Such studies may entail a scientific assessment of nutrient composition, the creation of novel products, and clinical trials to guarantee product safety and efficacy. For example, melatonin, developed under the auspices of Symphony Natural Health, has been demonstrated to act as an effective antioxidant, immune activator, and mitochondrial modulator when administered as a dietary supplement, which may confer benefits to human health (129). Several commercial entities are also engaged in environmental and health impact studies, to explore the mechanisms by which environmental factors affect health and how to reduce the adverse effects of environmental pollution on people’s health. For example, GlaxoSmithKline, Eli Lilly, and Abbott are reducing their carbon emissions (130). The aforementioned studies may encompass a variety of domains, including environmental monitoring, toxicological studies, and environmental health assessments. These endeavors can inform the formulation of environmental policy and planning, thereby safeguarding the health and wellbeing of individuals and the integrity of the natural environment. The overwhelming majority of companies have publicly declared their intention to reduce greenhouse gas emissions. This is achieved through the implementation of strategies such as the optimization of manufacturing and distribution processes and the sourcing of raw materials in an appropriate manner (131). The impact of scientific research conducted by commercial entities on public health is positive. This is due to the provision of superior medical, nutritional, and health products, the advancement of health education, and the promotion of disease prevention. Collectively, these factors contribute to an improvement in the population’s overall health.

It is also incumbent upon commercial entities to comply with the ethical and regulatory standards that govern scientific research. These standards are designed to ensure the research process’s safety, reliability, and impartiality. For example, in 1999, Merck & Co. initiated a large clinical trial on the anti-inflammatory drug rofecoxib (Vioxx) and misrepresented the results to hide evidence of rofecoxib’s cardiovascular toxicity. Subsequently, the company proceeded to disseminate the drug to healthcare professionals, furnishing them with deceptive data. This resulted in a considerable number of cardiovascular incidents that could have been averted in patients undergoing treatment with the drug (132).

#### 2.4.2 Marketing management

The marketing strategies of commercial entities profoundly impact on people’s health in several ways, including product promotion, consumer behavior, public health, and social responsibility. Some industries have implemented self-regulatory codes and industry standards to reduce the promotion of unhealthy products. Some food companies have initiated educational campaigns to inform consumers about the significance of healthy lifestyles. These companies have also pledged to reduce the prevalence of junk food advertisements targeting children while promoting healthy food options. On the one hand, businesses promote healthy eating and organic products through advertisements to guide consumers to make healthier dietary choices by highlighting their nutritional value and health benefits. On the other hand, commercial entities promote the benefits of exercise and regular health check-ups, which help to raise public health awareness and behavior. A considerable number of business entities provide support for public health through the implementation of corporate social responsibility programs. For example, this may entail providing financial support for health research, facilitating the implementation of health education programs, or promoting employee wellness initiatives. These initiatives have the potential to enhance public health to a certain extent. Some businesses and public health organizations have formed collaborative partnerships to promote healthy behavioral change by implementing social marketing strategies. For example, anti-tobacco campaigns and healthy eating promotions, as marketing management, influence public behavior and improve health outcomes.

The promotion of unhealthy foods, such as fast food, high-sugar drinks, and snacks, through advertisements and promotional campaigns is a common practice that targets children and adolescents. The consumption of these nutritionally deficient foods is significantly correlated with an increased risk of developing a range of chronic health conditions, including obesity, diabetes, cancer, and cardiovascular disease (133). Despite the existence of stringent regulations on tobacco advertising in numerous countries, tobacco companies persist in employing a multitude of marketing strategies that exert a significant influence on smoking behavior, particularly among adolescents. This has resulted in a considerable burden of health problems, including lung cancer, heart disease, and respiratory illnesses (134). The marketing of alcoholic beverages has been linked to adverse effects on public health, with a notable impact on adolescents. The abuse of alcohol is associated with a range of adverse health outcomes, including liver disease, gastrointestinal disorders, heart disease, neurological disorders, cancer, mental health issues, and an elevated risk of accidents (135). The relaxation of certain alcohol consumption restrictions in Poland in 2001, coupled with the proliferation of beer advertisements, resulted in a notable increase in alcohol-related mortality rates among the male population, from 13.9/100,000 in 2002 to 28/100,000 in 2017. Similarly, alcohol-related mortality among the female population rose from 1.7/100,000 in 2002 to 6.4/100,000 in 2017 (136). Furthermore, alcohol companies target younger age groups and females in their advertising, thereby normalizing alcohol consumption among younger individuals and women, which in turn leads to increased alcohol consumption and subsequent adverse effects on public health (34). Some companies may disseminate false or misleading information in their advertisements, inducing consumers to purchase products that do not align with the advertised effects. Such marketing strategies may result in consumers misinterpreting health information, which may influence their health decisions. The marketing of health products and nutritional supplements frequently overstates the efficacy of these products, leading consumers to believe that they can substitute for a healthy lifestyle and medical advice, which may pose health risks. The purchase of medicines online is becoming increasingly common. While these are purchased from websites that appear to be legitimate, the supply chains involved are often opaque, increasing the risk of buying counterfeit or substandard medicines. A 2013 study of pharmaceuticals from 19 countries revealed that 9.1% of tuberculosis medication samples lacked sufficient active ingredients or failed disintegration tests. The drug failure rate was 16.6% in Africa and 10.1% in India, which could result in a significant number of child deaths from diseases such as pneumonia (137).

## 3 Burden of neurosurgery diseases

The most prevalent neurosurgical disorders are TBI, stroke, ICH, and malignant brain tumors. These conditions have a profound impact on the quality of life and survival of patients. Treating these diseases necessitates the use of sophisticated medical equipment and technologies, as well as a substantial number of healthcare professionals. Conversely, the management of these diseases requires the implementation of long-term rehabilitation programs and the provision of continuous supervision. However, the availability of medical resources is constrained, necessitating difficult decisions on resource allocation by the government and medical institutions.

### 3.1 Traumatic brain injury

TBI represents a significant global public health concern, with over 50 million individuals worldwide affected by TBI annually, resulting in economic losses estimated at up to $400 billion (1, 2). TBI is the leading cause of death in young people and the predominant reason for death and disability at all ages in countries across the globe. Approximately half of the global population is estimated to experience a TBI at some point during their lifetime (1). In low- and middle-income countries, road traffic accidents represent the primary cause of TBI, with the majority of these injuries occurring in younger individuals. Conversely, in high-income countries, falls are the predominant cause of TBI, with the majority of these injuries affecting older individuals (138). China has the distinction of being the most populous country in the world and also has the highest number of patients with TBI. It is estimated that up to 900,000 individuals in China experience a TBI annually, with an incidence rate of approximately 55–64/100,000 (139). In the United States, there are approximately 2.8 million cases of TBI per year, with an incidence rate of 800/100,00. Of these cases, more than 69,000 result in death annually, leading to an estimated 5.3 million people living with disabilities (140). A primary brain injury frequently results in secondary damage to the brain, including a loss of cerebral compliance, tissue hypoxia, seizures, metabolic disorders, and neuroinflammation. These consequences ultimately lead to cell death (141). Such pathophysiological changes have the potential to result in a range of complications, including disability, dementia, epilepsy, stroke, neurodegenerative diseases, PTSD, depression, and other neuropsychiatric disorders (1, 142–146). A study revealed that 49% of disabilities resulting from TBI occurred in individuals of age 40–69 years, 25% occurred in individuals of age 70 years and above, 22% occurred in individuals of age 20–39 years, and 5% occurred in individuals younger than 20 years old (147). This disability can result in limitations in working or engaging in daily activities. Furthermore, it may lead to an increased need for ongoing medical care, rehabilitation, support, and services, which presents a significant challenge to individuals and families. It is estimated that 43.3% of individuals with TBI in the United States experience disability. Furthermore, nearly one in five hospitalized survivors do not return to work 1 year after their injury because of their disability, resulting in an economic loss of $51.2 billion. This represents the highest financial loss of all traumatic injuries (148).

### 3.2 Stroke

Stroke represents a significant global public health concern. It is estimated that 101 million individuals worldwide are afflicted with stroke, resulting in 6.55 million deaths (3). Stroke represents the most prevalent acute vascular incident, with cerebrovascular occurrences (including stroke and transient ischemic attacks) accounting for 45% of all acute vascular incidents, coronary vascular for 42%, and peripheral vascular for 9% (149). Overall, from 1990 to 2019, there was a 70.0% increase in the incidence of stroke, an 85.0% increase in the prevalence of stroke, a 43.0% increase in the number of stroke deaths, and a 32.0% increase in the number of DALYs due to stroke (3). However, the incidence and burden of stroke exhibit considerable variation between developing and developed countries. In high-income countries, the incidence of stroke is estimated to range from approximately 125–466 cases per 100,000 individuals. In contrast, in low- and middle-income countries, the incidence of stroke is estimated to range from approximately 15–281 cases per 100,000 individuals (150). Overall, the incidence of stroke is higher in high-income countries than in low- and middle-income countries. However, WHO estimates that low- and middle-income countries account for 85.5% of all stroke deaths worldwide and have a 7-fold greater loss of DALYs than high-income countries (150). The overall mortality rate from stroke in high-income countries is approximately 20%, whereas in low- and middle-income countries, the overall mortality rate from stroke is approximately 25–35%. Furthermore, the incidence of stroke tends to decrease in high-income countries, whereas in low- and middle-income countries, the incidence of stroke tends to increase. The incidence of stroke in high-income countries exhibited a notable decline, from 163/100,000 in 1970 to 94/100,000 in 2008. This represents a reduction of approximately 40% over four decades. Conversely, the incidence of stroke in low- and middle-income countries demonstrated a striking increase, from 52/100,000 in 1970 to 117/100,000 in 2008. This equates to an expansion of approximately 100% over the same period (150). Given the observed increase in the incidence of other acute vascular events in low- and middle-income countries, which mirrors the trend of stroke incidence, it can be postulated that this may be associated with changes in health and demographics in these countries. The prevalence of smoking, hypertension, diabetes, inadequate fruit and vegetable intake, high-salt diets, high-fat diets, and lack of physical activity in low- and middle-income countries represents a significant public health challenge, increasing the likelihood of acute vascular events (151, 152). As the most populous country in the world, China has the highest number of stroke patients globally, significantly exceeding the number of patients in the United States and the European Union (153–155). Since 2015, stroke has emerged as a significant public health concern in China, accounting for the greatest number of deaths and disabilities among non-communicable diseases. This trend represents a substantial challenge to Chinese citizens’ overall health and wellbeing (155). It is estimated that 17.8 million citizens in China experience stroke, resulting in 2.2 million disabilities and 2.3 million deaths (155). The incidence of stroke in China has increased significantly over the past decade, with a notable rise from 1,332/100,000 in 2010 to 2,610/100,000 in 2020. This alarming trend has placed a considerable burden on both individuals and families (155, 156). In 2019, the per capita hospitalization cost for stroke patients in China was renminbi (RMB) 9,800–20,100, representing an increase of 37–82% compared with 2010. This resulted in total hospitalization costs amounting to RMB 54.8 billion (155).

### 3.3 Intracerebral hemorrhage

ICH is typically defined as a primary cerebral hemorrhage, with hypertension being the primary cause. These hemorrhages typically occur in the basal ganglia, thalamus, cerebellum, and pons. The global incidence of ICH is estimated to be between 10 and 20 per 100,000 individuals, with an increase observed with advancing age (4). The incidence of ICH is higher in Asian and Black populations, with an estimated prevalence of approximately 50 cases per 100,000 individuals (157, 158). Despite the relatively low incidence of ICH compared to that of TBI and stroke, the prognosis for ICH is poor. The 7-day and 1-year mortality rates for ICH are as high as 31% and 59%, respectively (5). To date, no specific therapeutic options have been demonstrated to improve prognosis in patients with ICH. Consequently, the primary objective of treatment is to provide supportive care (159). Furthermore, only a small proportion of patients who survive for 1 year can live independently, indicating that ICH is not only a severe and fatal disease but also that the remaining survivors can impose a significant burden on family and national healthcare resources (160, 161). In addition to hypertension, cerebral amyloid angiopathy, smoking, alcohol consumption, hyperlipidemia, diabetes, and use of anticoagulants are identified as risk factors for ICH. These findings suggest that ICH can be prevented through changes in lifestyle and dietary habits (3).

### 3.4 Brain malignant tumor

Malignant brain tumors can be classified into two main categories: primary brain tumors and metastatic brain tumors. Gliomas represent the most prevalent primary malignant brain tumors, whereas brain metastases are predominantly derived from breast cancer, lung cancer, and melanoma. Gliomas represent over 80% of primary malignant brain tumors, with an incidence of approximately 7/100,000 and a tendency to increase with age (6). Although gliomas are less prevalent than TBI and stroke, they have a dismal prognosis and are costly to treat, frequently becoming a significant burden on individuals and families. Glioblastoma and astrocytoma are the most prevalent forms of glioma. Glioblastoma is most prevalent in individuals of age 75–84 years, with a 5-year survival rate of 5.4%. Conversely, astrocytoma is most prevalent in patients younger than 50 years old, with a five-year survival rate of approximately 44% (162). The specific etiology of gliomas remains unknown, although environmental and genetic factors have been identified as potential contributors to the disease’s development. It has been suggested that there may be a significant correlation between elevated socio-economic status and the prevalence of gliomas (163). The current standard of care for gliomas is surgical resection in conjunction with radiotherapy and chemotherapy. In select cases, adding electric field therapy or targeted therapy has been demonstrated to improve survival outcomes (164). The financial burden associated with temozolomide chemotherapy ranges from approximately $1,600 to $4,600 monthly. At the same time, the cost of radiotherapy can reach up to $9,000 per month, not including the expense of surgical intervention (165). Furthermore, other community members bear additional costs associated with the disease, including those related to disease progression. Consequently, gliomas impose a significant burden on families and healthcare systems. The incidence of brain metastases resulting from the growth of malignant tumors is approximately 9.6%. The majority of these metastases originate from lung cancer, accounting for 19.9% of cases, followed by melanoma (6.9%), renal cancer (6.5%), breast cancer (5.1%), and colorectal cancer (1.8%) (7). Patients with brain metastases resulting from a tumor may present with a range of neurological symptoms, including headaches, seizures, focal neurological deficits, cognitive impairment, memory loss, emotional abnormalities, hemiparesis, and gait abnormalities. These symptoms can significantly impact the quality of life of affected individuals. Once brain metastasis has occurred, the prognosis is poor, and treatment costs are likely to increase significantly (8). The majority of treatments for brain metastases are palliative and are unable to enhance patient survival rates. In the United States, the increase in healthcare costs associated with tumor brain metastases is estimated to be between $17,000 and $23,000 per month, primarily due to more expensive medications and longer hospital stays (166). Furthermore, indirect losses to the patient and family will result from the inability to work and the necessity for additional care from family members.

## 4 Implications for neurosurgery diseases

The field of public health has benefited from the effective application of political economics. Given the prominence of neurosurgery in public health, it is imperative that political economics dedicates sufficient attention to this field. The discipline of political economics can shed light on various aspects of neurosurgery, including disease prevention, treatment, research, and data analysis (Table 2). From a policy perspective, formulating appropriate educational initiatives could play a pivotal role in preventing certain neurosurgery-related diseases. Conversely, establishing hospitals, training of medical professionals, integrating medical services, and equal allocation of healthcare resources could facilitate more effective treatment of neurosurgery diseases. Furthermore, investment in drug research, technology development, and treatment innovation could enhance the prognosis of neurosurgery diseases. Data collection has the potential to be beneficial for research purposes, as well as to provide an accurate picture of the incidence of neurosurgical diseases, their treatment effects and recovery, and to offer a scientific basis for policymaking.

**Table 2:** Implications of political economics in neurosurgery diseases.

Implication	Neurosurgery disease	Description	References
Prevention	TBI	Since NAPL was implemented, the rapid decline in the incidence of road traffic accidents has greatly reduced the incidence of TBI in China	(167)
	TBI	TMHUL reduced motorcycle-related TBI by 33% and greatly reduced the length of hospitalization for TBI	(168)
	Stroke	BSCO reduced the incidence of smoking-related strokes	(169)
	Stroke and ICH	Government provides citizens with free medical kits containing healthy reading materials and measuring tools for salt and oil to prevent stroke and ICH	(169)
	Stroke	Health education in the community reduced the risk of stroke occurrence by 11.4%	(170)
Treatment	TBI and stroke	Industrialization and urbanization improved medical service centers and transportation facilities, leading to a decrease in the mortality and disability rates of TBI and stroke	(169)
	Stroke and ICH	Free medical check-ups for residents allow the patients of ICH and stroke could receive adequate attention and timely treatments	(169)
	Stroke	The establishment of stroke centers has increased the rate of implementation of intravenous thrombolysis, carotid endarterectomy, and carotid artery stenting and improved the prognosis of stroke patients	(171)
Research	Glioma	TTFields therapy elicits non-invasive anti-glioma therapeutic effectiveness	(182)
	Glioma	CAR-T therapy targeting B7-H3 shows its effectiveness in recurrent glioblastoma	(185, 186)
	Glioma	Vaccines, oncolytic virus, and many other immunotherapies may be potential modalities for glioma therapy	(187–190)
	Stroke	Combination pills containing aspirin, lisinopril, atenolol, and simvastatin can reduce the incidence of atherosclerotic heart disease and stroke, improve patient adherence, and significantly reduce healthcare costs and personal financial burdens	(191)
	TBI	Development of smart driving or smart answering of phone calls and sending text messages may help to reduce the incidence of traffic accidents and TBI	(192, 193)
Data	TBI	CDCPUS uses standardized case definitions and data collection methods to detect TBI, focusing on admission status and discharge outcomes	(194)
	Stroke	CSPPC provides free screening and treatment guidance for citizens over 40 years old, which helps to provide an epidemiological basis for the health authorities to assess the trend of stroke incidence	(195)
	TBI and stroke	A fall detector for the older adults project in Spain aims to provide detection and early warning of falls in the older adults which allows for the collection of fall-related TBI or stroke data	(196)
	Glioma	TCGA and CGGA are global cancer genome atlases which provide clinical gene sequencing data for a variety of tumors and gliomas so that researchers can analyze data to find suitable therapeutic targets	(198, 199)

BSCO, Beijing Smoking Control Ordinance; CAR-T, Chimeric antigen receptor T-cell; CDCPUS, Centers for Disease Control and Prevention of the United States; CGGA, Chinese Glioma Genome Atlas; CSPPC, China Stroke Prevention Project Committee; NAPL, National Alcohol Penalty Law; ICH, intracerebral hemorrhage; TBI, traumatic brain injury; TCGA, The Cancer Genome Atlas; TMHUL, Taiwan Motorbike Helmet Use Law; TTfields, tumor treating fields.

### 4.1 Prevention

It is imperative that political-economic decisions prioritize the prevention of neurosurgical diseases. The incidence of TBI can be reduced by implementing legislative and policy instruments, such as the establishment of strict traffic safety regulations, the enactment of workplace safety standards, and introducing protective measures for sports. In 2011, the Ministry of Public Security of China enacted the ‘National Alcohol Penalty Law’, which stipulates that all individuals driving under the influence of alcohol will be subject to administrative penalties upon being apprehended. Concurrently, regulatory authorities periodically administer alcohol tests to drivers to guarantee road safety, particularly during the Chinese New Year. As it is customary for Chinese people to gather for meals and consume alcohol during the Chinese New Year, the public security and traffic police have intensified their enforcement activities during this period, resulting in a notable decline in the incidence of driving under the influence. Subsequently, the precipitous decline in the incidence of road traffic accidents has led to a notable reduction in the incidence of TBI (167). In developed countries, the primary cause of TBI is falls, whereas in developing countries, road traffic accidents are the predominant cause of TBI. It would benefit other developing countries to consider adopting this practice in China. Furthermore, the implementation of protective measures is crucial for the prevention of TBI, including the use of helmets and seat belts. A case in point is the ‘Taiwan Motorbike Helmet Use Law’, enacted in 1997 in Taiwan, China. This legislation has been shown to reduce the incidence of motorcycle-related TBI by 33% and significantly decrease the length of hospitalization for TBI (168). In particular, using helmets and seat belts represents a highly cost-effective and efficient method of preventing TBI in some low- and middle-income countries, where infrastructure is often inadequate, and the number of motor vehicles is increasing. Furthermore, the implementation of efficacious public health policies can mitigate the prevalence of stroke and ICH. For example, the incidence of stroke and ICH can be controlled by reducing risk factors such as high blood pressure, diabetes, and high cholesterol through tobacco control, alcohol control, the promotion of healthy diets, and regular medical check-ups. Beijing has implemented one of China’s most stringent tobacco control regulations, the ‘Beijing Smoking Control Ordinance’, which represents a significant advancement in China’s efforts to combat tobacco use (169). The legislation in question prohibits smoking in all indoor settings and the majority of outdoor public spaces. Those who violate this law are subject to extremely severe penalties. Furthermore, the legislation prohibits the advertising of tobacco products in public spaces. These measures have resulted in a notable decline in the prevalence of smoking-related strokes. Nevertheless, in economically underdeveloped regions, implementing these measures encounters obstacles. On the one hand, local governments may require revenue from tobacco sources, and on the other hand, citizens are not adequately informed about the dangers of smoking. It is, therefore, imperative that a shift in the pattern of economic growth in these regions occurs, as well as improvements to the quality of national education. Although numerous risk factors for brain tumors remain poorly defined, the potential for reducing their incidence can be achieved by reducing known environmental risks (e.g., radiation exposure) and promoting healthy lifestyles. It would be beneficial for governments to provide financial support for further research into the risk factors for brain tumors.

It is recommended that the government and non-governmental organizations collaborate to enhance public education and awareness regarding preventing neurosurgical diseases. This should include promoting educational activities on healthy lifestyles and disseminating information about the risks associated with neurosurgical diseases and the protective measures that can be taken. It is possible to enhance the general population’s health awareness by implementing publicity initiatives within educational institutions, local communities, and workplaces. A randomized controlled trial for stroke prevention was conducted in three Chinese cities: Beijing, Shanghai, and Changsha (170). The trial provided health education in the intervention community, including guidance on adopting a healthy lifestyle, increasing exercise, limiting salt intake, quitting smoking and alcohol consumption, and information on the role of hypertension, diabetes, and coronary heart disease in stroke occurrence. Individuals at risk of stroke, such as those with hypertension or diabetes, were also instructed to undergo regular monitoring and treatment. The control community did not receive any form of intervention. Over 10 years, during which the intervention was implemented and long-term registration and follow-up were conducted, the risk of stroke occurrence was reduced by 11.4% in the intervention group. This finding suggests that health education is important in preventing stroke occurrence. Indeed, the financial burden associated with this intervention is relatively modest and can be extended to the majority of cities in nearly all countries. In particular, there is a significant opportunity for health education in economically underdeveloped areas where citizens lack awareness of risk factors such as hypertension, diabetes, hyperlipidemia, and smoking. The Shanghai municipal government provides its 8 million citizens with free medical kits containing educational materials and measuring tools for salt and oil annually (169). A diet low in salt and fat can help prevent strokes and ICH. While the cost of these items is not high, the significance of health education for citizens is considerable and beneficial for other countries and regions. In addition to stroke, other neurosurgical diseases can be prevented through a similar approach. For example, a nationwide educational initiative on the management of emergencies and appropriate responses at the scene of a TBI or stroke could significantly reduce mortality and disability rates by ensuring that injured individuals receive medical assistance promptly. In some countries and regions, child abuse represents a significant contributing factor to the incidence of TBI. Consequently, it is imperative to enhance parental awareness and education regarding the consequences of TBI and to facilitate the provision of shelters for abused children.

### 4.2 Treatment

Political economics is concerned with the equitable and efficient distribution of resources. This is of particular importance about the treatment and rehabilitation of neurosurgical diseases. The equitable distribution of medical resources, including emergency equipment, medicines, advanced imaging equipment, specialist medical teams, and rehabilitation facilities, is crucial for ensuring timely and efficient medical care for patients with neurosurgery diseases. This distribution should be balanced between urban and rural areas and regions of different economic levels to improve the prognosis of patients and reduce the burden on society. Since its reform and opening in 1978, China has undergone the most extensive industrialization and urbanization in human history. This has resulted in a significant increase in the urban population, from 170 million in 1978 to 770 million in 2015, with the urbanization rate growing from 117.9% to 56.1% (169). Healthcare services is more extensive in urban than rural areas, enabling urban residents to receive more efficacious treatment. Furthermore, the enhancement of medical service facilities and transportation infrastructure in urban areas has enabled residents to reach medical centers promptly, facilitating the administration of appropriate treatment in emergencies such as severe TBI and stroke. This has resulted in a notable reduction in the mortality and disability rates associated with neurosurgical diseases among residents. As evidenced by statistical data, in 2015, the per capita doctor ownership rate for urban residents in China was 3.72 per 1,000 individuals, while the rate for rural residents was 1.55 per 1,000 individuals (169). Large-scale urbanization represents the most direct means of enhancing the quality of healthcare for all citizens, a strategy that other developing countries may emulate to improve their healthcare systems. However, the unequal distribution of medical resources resulting from China’s Reform and Opening Up is also evident. Prior to the Reform and Opening Up, China’s healthcare resources were among the highest in the world in terms of average. However, following the Reform and Opening Up, many individuals lost their insurance coverage due to the introduction of market mechanisms, which directly resulted in a rapid deterioration in the equity of the healthcare system. The equitable distribution of healthcare resources represents a significant challenge within the political economy of socialist countries. It is incumbent upon national and local governments to develop sound healthcare policies that encompass diagnosis, emergency care, treatment, and long-term rehabilitation for patients with neurosurgical diseases. Policies must include provisions for targeted assistance to low- and middle-income groups to reduce delays in treatment due to financial constraints. This necessitates a comprehensive examination of medical resources, financial budgets, and social welfare. Luzhou in China has provided free medical check-ups for residents since 2016. The program includes various services, such as physical examinations, blood tests, X-rays, and more (169). Such a measure not only promotes the fair distribution of medical resources and ensures that patients can detect diseases promptly and receive effective treatment, but it also provides data for epidemiological research, which helps health departments formulate relevant policies. Statistical data from medical check-ups in Luzhou indicate that 17% of residents suffer from high blood pressure and 5.5% from diabetes. These figures suggest that this population may be at an increased risk of stroke and intracerebral hemorrhage and require further attention. However, it is important to note that this initiative’s replicability is limited. This is due, in part, to the substantial costs associated with providing free healthcare to the population, including the investment of labor, supplies, and financial resources. Additionally, there is necessary to ensure the availability of an adequate number of local healthcare resources. The feasibility of this approach may be limited in some economically less developed areas. Conversely, this measure may be more applicable in some economically developed or less populated areas.

Political-economic decisions must facilitate the development of policies that assist families and communities in caring for individuals with neurosurgical diseases. This can be achieved by providing the requisite financial subsidies, training, and psychological support, alleviating the burden on families. Furthermore, government-sponsored increases in pensions for the older adults and reimbursement rates for neurosurgery diseases have the potential to significantly reduce mortality and disability rates associated with these diseases. A comprehensive system of rehabilitation services for neurosurgery diseases, including medical, psychological, vocational, and social support, should also be established to facilitate patients’ reintegration into society and enhance their quality of life. For example, the establishment of stroke centers in China has provided new insights into the improvement of stroke treatment, the control of stroke morbidity, and the implementation of comprehensive post-stroke treatment (171). Stroke centers in China are classified into two categories: advanced stroke centers (hospital level: level III and above) and stroke prevention centers (hospital level: level II and above). The advanced stroke center engages in the regular implementation of pivotal stroke prevention and control technologies, actively advocates for establishing a two-way referral mechanism for stroke within the region, and strives to enhance the overall stroke prevention and control capacity within the region. The criteria for the establishment of a stroke prevention center include the establishment of a green channel for emergency multidisciplinary collaboration, the implementation of venous thrombosis techniques, participation in the construction of a regional graded stroke treatment network, and the implementation of stroke prevention and secondary prevention measures. The establishment of stroke centers has resulted in an increased rate of implementation of intravenous thrombolysis, carotid endarterectomy, and carotid artery stenting, as well as an improved prognosis for stroke patients. A 15% reduction in complication and mortality rates was observed among patients treated with thrombolytic therapy at stroke centers (171). Data from the United Kingdom indicate that the implementation of stroke unit care within routine clinical practice is associated with a reduction in mortality rates by approximately 25%. This finding is consistent with the results of systematic analyses of data from stroke unit trials (172). Enhanced imaging of the brain, cerebrovascular system, and heart will facilitate a more comprehensive understanding of the underlying mechanisms of stroke, the acute care required, and the secondary prevention strategies that can be employed. However, this increased understanding will also lead to increased hospitalization costs and a greater demand on hospitals’ equipment and staffing resources (173). Stroke centers are a common feature of economically developed countries. However, in some economically underdeveloped countries or regions, establishing stroke centers should be promoted to improve the prognosis of patients. In cases where the economic situation does not allow for the establishment of sufficient stroke centers, increasing the number of patients is an alternative option that can be considered to improve prognosis. The number of patients treated is a statistically significant factor in stroke outcomes. Hospitals with a higher volume of stroke patients tend to have an improved prognosis, particularly in terms of mortality, even if they are not designated as stroke centers (174).

### 4.3 Research

The field of political economics underscores the pivotal role of innovation and scientific and technological advancement in social advancement. It is recommended that the government allocate greater financial resources to support fundamental and clinical research initiatives about neurosurgical conditions. Furthermore, there is a need to facilitate the advancement of novel therapeutic modalities and technological advancements. The treatment of glioma represents a significant challenge in neurosurgery, largely due to the uncertainty surrounding its etiology and the generally unfavorable prognosis associated with this condition. At present, the primary treatment approach for gliomas is a combination of surgical resection, postoperative radiotherapy, and chemotherapy (175, 176). In recent years, the development of single-cell sequencing has led to a growing body of evidence indicating that the heterogeneity of gliomas and the tumor microenvironment play a significant role in tumorigenesis and progression (177–181). This has resulted in a gradual evolution of glioma treatment toward precision and individualized therapy. Recent studies have identified tumor treating fields (TTFields) therapy, targeted therapy, and immunotherapy as potential innovative treatment modalities for gliomas. TTFields represent an emerging non-invasive anticancer therapeutic modality that involves the transcutaneous delivery of low-intensity (1–3 V/cm), intermediate-frequency (100–300 kHz), alternating electric fields (the approach is also known as alternating electric field therapy) that exert biophysical force on charged and polarizable molecules known as dipoles (182). TTFields elicit therapeutic effects by disrupting various biological processes, including DNA repair, cell permeability, and immune responses, thereby demonstrating a wide range of mechanisms of action.

It has long been assumed that the brain is immune privileged due to the presence of the blood–brain barrier. Nevertheless, recent studies have demonstrated that immune cells are indispensable for the functional maintenance and repair of the CNS (183). Chimeric antigen receptor T-cell (CAR-T) therapy represents a form of adoptively transferred T cell therapy that employs engineering techniques to induce T cells to express receptors of tumor-specific antigens on their surface. This receptor can bind to the corresponding antigen on the surface of tumor cells, thereby initiating a cytotoxic response (184). A CAR-T therapy targeting B7-H3, which is upregulated in glioma, has demonstrated efficacy in the treatment of recurrent glioblastoma (185, 186). Vaccines may represent a promising avenue for glioma immunotherapy in the future. The simplicity of vaccine production allows for the creation of individualized vaccines, which can be tailored to treat different gliomas in different patients. This addresses the heterogeneity issue inherent to gliomas (187). The recently developed lipid particle aggregate technique has the potential to effectively present tumor-derived mRNAs to immune cells, suggesting that vaccines for glioma may offer a promising avenue for immunotherapy (188). Furthermore, the oncolytic virus has demonstrated considerable promise in the immunotherapy of glioma (189). Nevertheless, further research is required to substantiate the efficacy and safety of these treatments over the long term (190).

These recent findings illustrate the encouraging progress being made in the field of glioma research. It is imperative that research funding be allocated to address the specific needs of populations with high morbidity, high risk, low survival, and high disability. Furthermore, there is a pressing need for active follow-up on the latest international research advances to promote interdisciplinary collaboration in neuroscience, immunology, epidemiology, public health, economics, and physics. This will facilitate a more comprehensive understanding of and response to the complex problems in neurosurgery diseases. Active involvement in international neurosurgery disease prevention and treatment programs, coupled with the sharing of experience and research findings with other countries, can promote and apply novel technologies and methodologies, thereby enhancing the efficacy of neurosurgery disease prevention and treatment. It would be beneficial to examine the successful experiences of other countries in the prevention, treatment, and rehabilitation of neurosurgical diseases. These experiences could then be implemented and improved in light of the actual situation to reduce the incidence of neurosurgical diseases, the mortality rate, and the disability rate. The combination of aspirin, lisinopril, atenolol, and simvastatin in a single pill has been demonstrated to reduce the incidence of atherosclerotic heart disease and stroke. Furthermore, it has been shown to improve patient adherence and significantly reduce healthcare costs and personal financial burdens compared to existing treatments (191). This result has been validated in five countries with varying degrees of economic development: China, India, Mexico, Nigeria, and South Africa. This suggests that the potential for replication in majority of the other countries is high.

The use of mobile phones and other forms of communication while driving has been identified as a significant contributing factor in road traffic accidents (192). Statistical evidence indicates that up to 90% of road traffic accidents are attributable to driver error (193). It can be reasonably deduced that the advancement of intelligent driving and the ability to respond to phone calls and send text messages more efficiently may contribute to a reduction in the number of traffic accidents, subsequently leading to a decline in the prevalence of TBI. Implementing this solution necessitates enhanced collaboration between various stakeholders, including health professionals, urban planners, information technology experts, and communication service providers, to mitigate potential adverse effects. Numerous challenges must be addressed before automated driving technology can be fully realized. These include the potential for cyber-attacks, the integration with existing road networks, and the need for reliable communication coverage. There is still a considerable distance to traverse before this objective is attained.

### 4.4 Data

The field of political economics places a significant emphasis on the role of data in informing decision-making processes. The establishment of a data collection and monitoring system for neurosurgery diseases, including electronic medical records, social media, patient summaries, genomic and drug data, clinical trials, telemedicine, mobile apps, behavioral and socio-economic indicators, and other relevant data sources, provides an accurate and comprehensive picture of the incidence, treatment effects, and recovery outcomes of neurosurgery diseases. Furthermore, it offers a scientific basis for evidence-based policymaking. The application of big data and artificial intelligence technologies has the potential to enhance the efficacy of neurosurgery disease prevention and treatment strategies and elevate the quality of decision-making processes.

It is important to note that epidemiological data reported by countries may differ due to differences in epidemiological study methods, such as case selection and admission policies. Therefore, standardizing international epidemiological study methods and reporting is of great interest. The Centers for Disease Control and Prevention (CDCP) of the United States has been employing standardized case definitions and data collection methods to detect TBI, focusing on admission status and discharge outcomes (194). China is deficient in reliable data regarding the incidence of TBI and the associated healthcare burden is considerable (1). In this regard, it is imperative that epidemiological testing in China and other developing countries be strengthened to provide reliable data that can inform the development of policies. In 2011, the Chinese government established the China Stroke Prevention Project Committee, which provides free screening and treatment guidance for citizens of age above 40 years. This initiative helps to establish an epidemiological basis for the health authorities to assess the trend of stroke incidence and to enhance treatment guidance for patients (195). The European Union has initiated a series of significant data projects to assemble a diverse array of data types, including medical practitioner records, hospitalizations, pharmaceutical prescriptions, and laboratory and radiological analyses. These data are being aggregated to construct comprehensive national data warehouses. These include the ‘Decision Support for Health Policy and Planning’ project. The ‘Methods, Models and Technologies based on Existing Health Care Data’ (DEXHELPP) project, the eHealth project, the ARNO observatory project, and the Hospital Episode Statistics project are also relevant in this context (196). The objective of the fall detector for the older adults project in Spain is of 2-folded: first, to facilitate the detection and early warning of falls in the older adults, and second, to enable the collection of data about fall-related TBI or strokes. Furthermore, the project aims to provide timely assistance services for the older adults (196). The Cancer Genome Atlas (TCGA) and the Chinese Glioma Genome Atlas (CGGA) are global cancer genome atlases initiated by the United States and China, respectively. They provide clinical gene sequencing data for various tumors and gliomas, which researchers can analyze to identify potential therapeutic targets (197–199). The advent of medical big data has facilitated international scientific collaboration, and may further identify cancer driver genes and therapeutic targets with the help of artificial intelligence and machine learning. In particular, developing their own big data centers and detection systems is a significant challenge for low- and middle-income countries. Nevertheless, utilizing internationally accessible open-access databases can offer a degree of reference value about the epidemiology and genomics of neurosurgical diseases within the country. Furthermore, the collection of large data sets raises several ethical concerns. For example, the collection of big data may result in the leakage of personal privacy, the commercialization of data, and the exposure of genetic data of its citizens to hostile countries, which may result in gene wars. Both national governments and international organizations must reach a consensus on the utilization of data, to attain a specific degree of confidentiality, security, and adherence to ethical and legal standards.

## 5 Current challenges and future directions

Political-economic decisions in the healthcare filed are confronted with many challenges, including the allocation of resources, the impact of globalization, the scientific basis of policy, strategic planning, the protection of privacy, and the resolution of ethical issues. It is of the utmost importance to determine how to confront these issues and propose solutions to advance political economics in health. Improved communication between policymakers, healthcare workers, economists, and other professionals is essential. The decision-making process in political economics about healthcare entails the consideration of trade-offs and choices among a multitude of complex interests, constrained resources, and evolving social environments. This inherently places significant demands on policymakers.

### 5.1 Resource

It is often the case that resources are limited, and governments must balance and allocate them among several areas, such as healthcare, education, and infrastructure. This can result in underfunding in the health sector. Determining which health problems or diseases should be prioritized for resources is a complex issue that involves several factors, including epidemiological data, societal preferences, and political pressures. Moreover, the involvement of diverse stakeholders, including pharmaceutical companies, insurance companies, healthcare providers, and patient groups, introduces the potential for varying objectives and priorities, possibly leading to conflicts of interest in the policymaking process. Interest groups may exert influence over decision-making processes through means such as lobbying and the provision of political contributions, which may result in the formulation of policies that reflect the interests of specific interest groups to a greater extent than the overall health interests of the public. It is not uncommon for tobacco companies to utilize financial resources to contest legislative measures, which can present a significant obstacle in low- and middle-income countries. British American Tobacco offers financial incentives to politicians, civil servants, and relevant policymakers in Africa and Asia, including through the use of bribery, to influence the policies of these countries in a manner that is favorable to the interests of the tobacco industry (200, 201).

Currently, neurosurgical diseases, including TBI, stroke, ICH, and brain tumors, represent a significant public health concern, imposing a considerable burden on individuals and healthcare resources. These conditions warrant sufficient resources to enhance patient outcomes and facilitate further scientific inquiry. The government must allocate a greater proportion of resources toward enhancing healthcare infrastructure, including the construction of hospitals, the integration of healthcare services, the provision of medical training, the recruitment of medical professionals, and the development of telemedicine. Establishing stroke centers in China has provided new insights into enhancing stroke treatment, controlling stroke morbidity, and implementing comprehensive post-stroke care. This has led to increased implementation of intravenous thrombolysis, carotid endarterectomy, and carotid artery stenting, as well as improved prognoses (171).

Stroke centers are a common feature of the healthcare infrastructure in developed countries. However, in some economically underdeveloped countries or regions, establishing stroke centers should be promoted to improve the prognosis of patients. In constrained resources, ensuring equitable access to health services for all social groups represents a significant challenge, particularly in rural and remote areas. Those in impoverished communities in economically underdeveloped regions frequently lack access to sufficient health services and resources, perpetuating a vicious cycle of health disparities. These areas frequently lack the requisite medical infrastructure and professional staff, thereby impeding residents’ ability to access timely and quality medical services. The Chinese government has implemented a series of measures to facilitate the transfer of medical resources to the grassroots level. One such measure is the provision of subsidies and preferential treatment to primary healthcare personnel in rural areas concerning promotion and title evaluation. These measures have enhanced the mobility of medical professionals to remote areas, and to a certain extent, elevated the standard of medical care in the region, which is worthy of emulation by many developing countries. Furthermore, implementing community health programs, particularly in rural and remote areas, can enhance population health through mobile medical services, health education, professional flow, and preventive measures. For example, the government can promote innovative medical service models such as family doctor systems, telemedicine services, and short-term medical services, among others, to meet the diverse medical needs of the population, particularly in remote and resource-poor areas (119–121).

Through policy reforms, governments can invest in the primary healthcare system, expand health insurance coverage, provide subsidies and financial assistance, reduce the healthcare burden of poor groups, and ensure that all people can afford basic medical services, including prevention, diagnosis, and treatment. This will not only enhance overall health but also mitigate the considerable financial burden associated with serious illness. The Chinese government has mandated that medical students complete 3 years of standardized residency training after graduation and before practicing medicine. This has led to significant improvements in the overall standard of healthcare in China and a reduction in the excessive disparities in healthcare services across different regions (202). In particular, some complex subspecialties, such as neurosurgery, necessitate a minimum of two to 4 years of specialized training.

### 5.2 Globalization

National boundaries do not confine the challenges posed by infectious diseases and environmental pollution and cannot be addressed by the efforts of a single country. Instead, these issues demand a collaborative approach that transcends the limitations of individual nation-states, necessitating international cooperation and coordination. Historically, the rapid transnational spread of infectious diseases, as evidenced by the Spanish Flu in 1918, the SARS epidemic in 2003, the Ebola epidemic in 2013, and the COVID-19 epidemic in 2019, has underscored the necessity for close international collaboration in disease surveillance, information sharing, and resource coordination to effectively address the challenges posed by these global health threats (203–206). This encompasses not only the prevention, control, and management of disease but also the facilitation of scientific research collaboration, the transfer of technology, and the provision of humanitarian assistance to affected countries. However, there are significant discrepancies in health policies, resources, and capacities among different countries, further complicating global health governance. Effective global health governance necessitates the coordination and transnational collaboration of international organizations to facilitate the sharing of information, the deployment of resources, and technical cooperation. In instances of global epidemics or health crises, wherein vaccines and medicines are in short supply, numerous countries prioritize meeting the needs of their citizens, which can result in challenges about international distribution. For example, during the COVID-19 epidemic, some countries adopted a policy of ‘vaccine nationalism’, prioritizing the procurement and stockpiling of vaccines, which made it challenging for other countries, particularly low-income countries, to obtain sufficient quantities of vaccines.

To address these issues, countries must enhance their coordination and collaboration with international organizations and other nations. This entails the sharing of information and resources, as well as the development of unified response strategies. It is recommended that technology transfer and international cooperation be employed to enhance the production capacity of drugs and vaccines in developing countries, thereby ensuring the stability of the global supply chain. The global distribution mechanism for vaccines must be enhanced and reinforced to guarantee the equitable distribution of vaccines and medicines to all countries by their respective needs, with particular attention to low-income countries and vulnerable populations. In the global health crisis context, it is imperative to relax patent restrictions and implement reasonable drug pricing policies to ensure that all countries have access to the necessary medical resources. In essence, global health governance necessitates that countries transcend their national interests and collaborate earnestly to confront shared health challenges. Only through collective action on the part of the international community that equity and security in global health can be achieved.

### 5.3 Scientificity

The formulation of health policy is contingent upon the availability of scientific evidence. This evidence can inform policy decisions by providing insights into disease prevalence trends, the efficacy of preventive measures, the longevity of treatments, and the potential for health problems. Using scientific evidence guarantees the effectiveness and scientific rigor of policies. For example, epidemiological studies, clinical trials, and public health surveys can furnish policymakers with data that can inform the formulation of prevention and control strategies. Nevertheless, in the context of actual decision-making, politicians frequently encounter pressures from diverse interest groups, political parties, and the public. These pressures often compel them to negotiate compromises by political interests, irrespective of scientific evidence, leading to the adoption of policies that are not necessarily aligned with scientific principles. For instance, during the promotion of vaccines, governments may encounter pressure from anti-vaccine groups. These radical anti-vaccine groups disseminate misinformation and engage in intimidation tactics against medical professionals to influence the development and implementation of vaccination policies, distorting and exaggerating the side effects of vaccines through publicity (207). It is also the case that commercial factors can influence the development of health policy. For example, pharmaceutical companies and medical device manufacturers may exert influence over policy through lobbying activities that prioritize the promotion of certain medicines and devices over other potentially more effective or cost-effective options. In 1999, Merck & Co. initiated a large clinical trial on the anti-inflammatory drug rofecoxib (Vioxx) and misrepresented the results to conceal evidence of rofecoxib’s cardiovascular toxicity. Subsequently, the company vigorously promoted the drug to healthcare professionals, furnishing them with deceptive data regarding its efficacy. This resulted in a considerable number of cardiovascular incidents that could have been averted in patients undergoing treatment with the drug (132). Furthermore, the lack of definitive information regarding many health issues (e.g., emerging infectious diseases) and the necessity for policymakers to respond expeditiously without comprehensive data render decision-making more challenging.

To address these issues, it is necessary to enhance communication between the scientific, medical, public health, political-economic, and policymaking communities, and increase transparency and public participation. It is recommended that independent scientific experts’ advisory boards be established to provide policymakers with direct access to the most recent scientific advice. Furthermore, the evidence derived from scientific research should be considered deliberately in policymaking. Moreover, it is imperative that science communication and training for policymakers be provided to enable them to comprehend scientific data and findings more effectively. This would enhance their scientific literacy, facilitating a more nuanced understanding, analysis, and application of scientific evidence in the context of policy formulation. Finally, it is essential to guarantee transparency throughout the policy formulation process. It is of the utmost importance that the scientific evidence be made public to provide the general public with a clear understanding of the rationale behind the policy in question, as this is a fundamental aspect of the scientific process. This transparency is essential to fostering greater acceptance of the policy. Conversely, a public participation mechanism should be established to allow the public and relevant stakeholders to express their views and suggestions during the policy formulation process. This would enhance the democratic and inclusive nature of the policy.

### 5.4 Planning

Those engaged in the political process and policy formulation tend to prioritize short-term, visible outcomes to secure the electorate’s support. However, this approach often results in disregarding scientific recommendations that necessitate long-term investment to achieve meaningful impact. A significant challenge in the health sector is the discrepancy between the necessity for long-term investment and planning and the tendency of policymakers to prioritize short-term outcomes. Many chronic diseases (e.g., heart disease and diabetes) and health problems (e.g., obesity, mental health) require long-term prevention and management strategies that cannot be made effective in the short term. Diabetes, hypertension, high blood pressure, and smoking are frequently identified as risk factors for stroke and ICH. Given the tendency of politicians to prioritize short-term outcomes, long-term public health initiatives (such as those aimed at preventing and controlling chronic diseases and providing health education) may not receive the necessary resources and attention, resulting in slow progress in the prevention and control of stroke and ICH. This makes it challenging to achieve the desired results. It is, therefore, imperative that national-level long-term public health strategies be formulated and the long-term health goals and development routes be clarified to ensure the continuity and stability of public health policies. For example, an intervention trial for stroke prevention was conducted in China in Beijing, Shanghai, and Changsha (170). A 10-year intervention and long-term registration and follow-up demonstrated an 11.4% reduction in the risk of stroke in the intervention group, which illustrates the efficacy of long-term health management.

Furthermore, the construction and upgrading of public health infrastructure (e.g., hospitals, clinics, laboratories) necessitates long-term investment and planning, rendering it unfeasible to complete such projects within a short timeframe. This necessitates that successive governments reach a consensus on long-term healthcare investment. In capitalist countries, governments elected under a democratic electoral system may espouse disparate political stances and have divergent interest bases, which can give rise to potential policy contradictions when governments change. In a socialist system, this contradiction is mitigated due to the state’s macrocontrol. To illustrate, numerous environmental regulations were eased during the Trump administration, whereas the Biden administration re-engaged with the Paris Agreement and proposed a comprehensive green infrastructure initiative (42, 43). Therefore, a special public health fund should be established across the different administrations to ensure financial security for long-term public health programs. This would prevent interruption or cutbacks in regular health services due to changes in administration.

In addition, public health emergencies (e.g., infectious disease outbreaks) require a rapid response to immediately restrain the spread of the epidemic and protect public health. In a public health emergency, the government typically allocates a significant portion of its resources to the emergency response, including the procurement of anti-epidemic materials and the construction of temporary medical facilities. Such a diversion of resources may consequently result in a reduction in the resources allocated to routine health services, which could adversely impact the quality and accessibility of daily healthcare services. In emergency situations, those responsible for formulating policy tend to prioritize implementing short-term measures that can produce immediate results. This approach, however, often results in the neglect of public health policies that require long-term perseverance and investment. Consequently, there is a lack of continuity and stability in the overall development of the public health system. The question of how to respond to public health emergencies while maintaining continuous investment in and managing daily health services is complex, requiring careful balancing of competing priorities. To effectively address the potential for sudden emergencies, it is essential to consider a range of factors, including the government’s capacity to respond, the allocation of public health resources, the ability to administer vaccinations, the robustness of the health system, the population’s level of preparedness, and the availability of specialized medical professionals (208). To address this issue, it is necessary to implement integrated assessment tools and long-term strategic planning. To ensure scientific and comprehensive decision-making, it is essential to combine the economic, health, and social benefits to assess the effects of policies across multiple dimensions. The aforementioned tools include cost–benefit analysis, neutrosophic goal programming models, Markov modeling, and methods such as cost-minimization, cost-effectiveness, and cost-utility (209–212). Furthermore, cost–benefit analysis can be employed to evaluate the relative merits of alternative policy options in terms of their immediate and long-term consequences. The Rapid Urban Health Security Assessment Tool enables local governments to effectively evaluate the strengths and weaknesses of their health systems, develop long-term plans, and enhance their capacity to respond to sudden and unexpected emergencies (213). These approaches enable policymakers to balance responding to short-term political pressures and pursuing long-term public health benefits.

### 5.5 Privacy and ethics

The implementation of novel technologies in healthcare, including gene editing and artificial intelligence, holds immense promise. Yet, it is also accompanied by significant ethical, privacy, and regulatory challenges. The process of gene editing entails the modification of the human genome, which may result in unintended consequences for future generations. In particular, embryonic gene editing has given rise to concerns about the potential for social injustice in the context of ‘designer babies’. In 2018, Chinese scientist He Jiankui announced the birth of the world’s first genetically edited baby. This event immediately caused a global uproar and triggered a worldwide ethical controversy and condemnation of human gene editing (214). Ultimately, He Jiankui was found guilty of practicing medicine illegally and sentenced to 3 years imprisonment (215). The protection of genetic data is a significant concern, as the leakage of genetic information can result in discriminatory practices and violations of individual privacy. The rapid development of gene editing technology may render the existing legal and regulatory framework inadequate in addressing the evolving landscape of this field. Consequently, there is a need for the formulation of new regulatory policies and ethical guidelines. The transparency and fairness of artificial intelligence algorithms are pivotal considerations. It is incumbent upon policymakers to guarantee that artificial intelligence systems are impartial and that their decision-making processes are transparent and auditable. Artificial intelligence and big data must safeguard patient privacy and prevent the misuse and leakage of data when processing vast quantities of healthcare data. The accelerated advancement of artificial intelligence and big data necessitates the establishment of novel regulatory structures to guarantee their security and efficacy while preventing excessive regulation that impedes innovation.

Furthermore, certain health-related decisions give rise to ethical considerations, including those about euthanasia and organ transplant allocation. It thus falls upon policymakers to achieve a balance between ethical and practical considerations. The topic of euthanasia has been a source of interest throughout history, from its earliest discussions in classical philosophy to the present day. Francis Bacon held that a physician’s responsibility extended beyond the mere restoration of health, encompassing the alleviation of suffering as well. Indeed, this perspective aligns with the contemporary conceptualization of euthanasia. Augustus Cæsar expressed a lifelong desire to be euthanised. The practice of euthanasia gives rise to several ethical considerations about the right to life. Those who advocate for euthanasia maintain that it upholds the autonomy and dignity of the patient. Conversely, those who oppose euthanasia argue that it violates the principle of ‘do no harm’ within the context of medical ethics and the patient’s right to informed consent. It is the responsibility of policymakers to establish a balance between the protection of patient autonomy and the safeguarding of life. This can be achieved by establishing clear legal and ethical guidelines to ensure that euthanasia is carried out under strict conditions. As an example of a country that has legalized euthanasia, Belgium did so in 2002, making it one of the first countries in the world to do so. Indeed, euthanasia and other end-of-life practices were prevalent in Belgium prior to the legalization of euthanasia. Statistical evidence indicates that euthanasia may account for up to 4.5% of all deaths (216). The aforementioned pervasive social acceptance contributed to the legalization of euthanasia. Similarly, the euthanasia legislation enacted in Spain is comparable. As indicated by the survey results, 77.5% of respondents expressed support for the implementation of a law regulating death with dignity (217). From a legal standpoint, the law on euthanasia recognizes a person’s subjective right to die. In the context of active euthanasia, two key elements must be established: the individual’s subjective wish to die and the motivation of the participant, which should be driven by compassion and the desire to alleviate the patient’s suffering to facilitate a peaceful death.

Furthermore, the distribution of organs for transplantation raises questions of fairness and efficiency. The question of equitably allocating organs to patients who need them most with limited organ resources is a complex ethical issue. It is incumbent upon policymakers to establish a fair and transparent organ allocation system that considers several factors, including the severity of the patient’s condition, waiting time, and the chance of success. This will ensure that the allocation process is fair and preclude the sale of organs for commercial purposes. To illustrate, Hong Kong’s organ allocation system is founded upon equality and the optimization of benefits (218). The principle of equality considers the length of time a patient has been waiting for a transplanted organ, as well as the patient’s age, without prejudice based on ethnicity, sex, income level, and geography. Benefit maximization considers the success rate of organ transplantation. Additionally, if a patient awaiting transplantation has previously donated an organ, that patient’s priority for obtaining a transplanted organ is increased. This principle of mutual benefit is also observed in countries such as the United States, Australia, and Israel. However, when implementing such incentives, it is essential to consider public attitudes and acceptance, which are perceived differently by citizens of diverse cultural backgrounds or ethnicities. For instance, surveys indicate that 71% of Canadians support financial incentives to promote organ donation, compared to less than 20% in the United States (219, 220). In East Asia, the Confucian cultural tradition has historically strongly emphasized the family unit. Therefore, this model of mutually beneficial incentives requires a familial framework to be more effectively implemented. Potential strategies include providing financial assistance for family funerals, bestowing medals upon family members, or offering other family members priority for organ transplants (221).

To adequately address these issues, it is essential to implement a multi-party approach, ensure transparent decision-making processes, improve legal and regulatory frameworks, guarantee the protection of privacy and data security, and provide comprehensive ethics education and training. First, establishing independent ethics committees is recommended to assess the implementation of novel technologies, such as gene editing and artificial intelligence, and ascertain their alignment with ethical standards. Second, it is recommended that there be an increase in public participation and discussion to make the decision-making process transparent and enhance public understanding and support for new technologies. Third, the timely updating and formulation of relevant laws and regulations by the development of new technologies could ensure the legality and safety of their application. Moreover, international collaboration should be enhanced, and global ethical and regulatory standards should be established to collectively address the challenges posed by new technologies. Moreover, adopting advanced data encryption and anonymization technologies is essential to safeguard the privacy of genetic and medical data. Rigorous data usage norms must be established to guarantee that medical data are utilized within the confines of legal authorization and to deter any potential misuse or leakage. Finally, medical practitioners must receive ethical education and training to enhance their understanding of and ability to address ethical issues in the application of new technologies. It is imperative to enhance public awareness of emerging technologies, such as gene editing and artificial intelligence, and to foster a stronger social consensus through scientific publicity and education.

## 6 Concluding remarks

At present, the global public health sector is confronted with several significant challenges. The most effective way to address these challenges is through a collaborative approach involving political decision-makers, healthcare professionals, and economists. The political economy of health has significant implications for several other fields, including economics, welfare, the environment, food and drug safety, pollution emissions, occupational safety, the quality of medical services, consumer rights, public health policy, healthcare policy, scientific research, and marketing management. These fields play an important role in public health. Given the significant impact of neurosurgery on public health, particularly in the context of TBI, stroke, ICH, and brain tumors, policymakers must prioritize adequate attention to this field. These conditions not only impose a considerable burden on family and societal healthcare resources but also present a significant challenge to the sustainability of the healthcare system. This review examines the influence of political-economic factors on public health and provides an overview of the burden of common neurosurgical diseases (TBI, stroke, ICH, etc.). Furthermore, the review addresses the impact of political economics on individual and societal healthcare resources and the implications of political economics for neurosurgery diseases, current challenges, and future directions. We hope this review will facilitate the integration of political economics, public health, and neurosurgery, thereby providing assistance in policy formulation and improving the prevention and diagnosis of neurosurgery diseases and the overall health of the population.
